# The actress was not on the balcony: testing the Pseudorelative-First Hypothesis in Spanish

**DOI:** 10.3389/fpsyg.2025.1546432

**Published:** 2025-03-05

**Authors:** Marta De Pedis, Adam Zawiszewski, Itziar Laka

**Affiliations:** Department of Linguistics and Basque Studies, University of the Basque Country (UPV/EHU), Vitoria-Gasteiz, Spain

**Keywords:** Pseudorelative-First Hypothesis, attachment, language processing, syntax, self-paced reading

## Abstract

Strategies for attachment resolution in double-antecedent relative clauses have been widely studied since the late 1980s, when a seminal study by Cuetos and Michell revealed that the principles of Late Closure and Minimal Attachment were met in some languages but not in others. These principles predicted a universal preference for low attachment whereas several studies obtained a high attachment preference in Spanish. Since then, high attachment preference has been reported in a variety of languages and with different methods. There have been several attempts at explaining high attachment preference, but none have succeeded. In 2014, the Pseudorelative-First (PR-First) Hypothesis was proposed: it claims that pseudorelative clauses (PRs) are the reason why some languages reveal a preference for high attachment. In this paper, we test the PR-First Hypothesis by means of two self-paced reading experiments in Spanish. Results (reading times and accuracy scores) show an overall preference for HA regardless of PR availability, indicating that the PR-First Hypothesis cannot account for the variation in attachment preferences found in the literature.

## 1 Introduction

The Pseudorelative-First Hypothesis (henceforth, PR-First) (Grillo and Costa, 2014) was proposed in order to account for the inter- and intra-linguistic variation in attachment preferences in sentences of the type *NP1 of NP2* followed by a relative clause (RC) [see (1a, b, c)]:

(1)
  a. Someone shot the maid of the actress that was on the balcony.
  b. Low Attachment (LA):
    Someone shot [_DP_ the maid [_PP_ of the actress_i_ [_RC_ that_i_ was on the balcony]]].
  c. High Attachment (HA):
    Someone shot [_DP_ the maid_i_ [_PP_ of the actress] [_RC_ that_i_ was on the balcony]].

**Figure 1:**
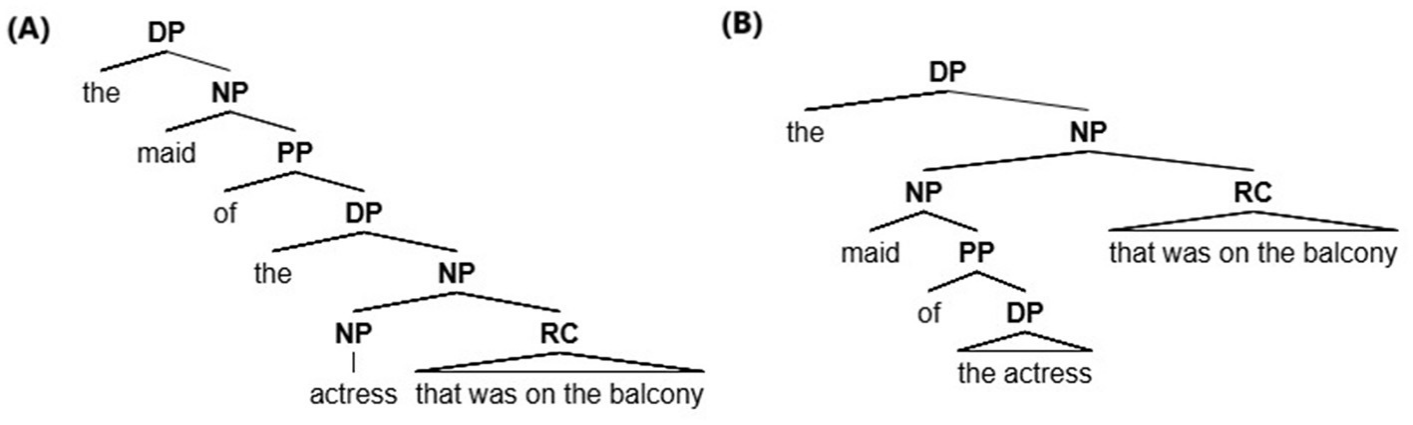
The syntactic representation in **(A)** shows Low Attachment (LA) and the syntactic representation in **(B)** shows High Attachment (HA).

The example in (1a) is structurally ambiguous: the relative clause (henceforth RC) can attach to the NP immediately preceding it (*the actress*) and the sentence is interpreted as having the actress on the balcony, a parsing option known as “Low Attachment” [henceforth LA, see (1b) and Figure 1A], because the relative clause attaches to the lowest syntactic node. Alternatively, the relative clause can link to the first NP (*the maid*), and the sentence is interpreted as having the maid on the balcony, a parsing option known as “High Attachment” [HA, see (1c) and Figure 1B], because the RC attaches to the higher node.

Faced with syntactic ambiguities like (1), Frazier (1979) proposed that the parser makes decisions based on two general economy principles:

(I) *Minimal Attachment: Attach incoming material into the phrase-marker being constructed using the fewest nodes consistent with the well-formedness rules of the language under analysis*.
(II) *Late Closure: When possible, attach incoming material into the phrase or clause currently being parsed*.

Frazier (1979) thus predicted a universal preference for LA in sentences like (1a) [that is, a preference for the interpretation in (1b)]. However, Cuetos and Mitchell (1988) revealed that Spanish speakers had a consistent preference for HA, preferring the interpretation in (1c). In the following decades, a growing body of literature further confirmed these findings for Spanish (Carreiras and Clifton, 1993, 1999; Cuetos et al., 1996; Gibson et al., 1999; Gilboy et al., 1995; Igoa et al., 1998; Mitchell et al., 1990) and extended them to other languages like Italian (De Vincenzi and Job, 1993, 1995), Portuguese (Maia and Maia, 2001; Miyamoto, 1998; Ribeiro, 1998, 2005) and French (Frenck-Mestre and Pynte, 2000; Zagar et al., 1997) among others. Moreover, attachment preferences were found to vary depending on methods used leading to a pattern of results that defied explanation, as discussed in the review provided by Grillo and Costa (2014).

The Pseudorelative-First (PR-First) Hypothesis (Grillo and Costa, 2014) claims that HA preference results when the attaching clause is not a RC but a Pseudorelative (henceforth PR). The Hypothesis states that:

*When PRs are available, everything else being equal, they will be preferred over RCs*.

The authors argued that previous research had overlooked the possibility of a Pseudorelative (PR) reading in sentences like (1a). Pseudorelatives are string identical to RCs but differ in structure and meaning; crucially for the hypothesis, some languages allow them (i.e., Spanish) while others do not (i.e., English). PRs describe events or situations (Cinque, 1992; Grillo and Costa, 2014). Consider the following examples from Spanish:

(1) *Llamé al hijo del panadero que corr*í*a en el parque*. I called the son of the baker that was running in the park.
(2) *Vi al hijo del panadero que corr*í*a en el parque*. I saw the son of the baker (that was) running in the park.

Example (2), like example (1a), is ambiguous between a LA interpretation [as in (1b)] and a HA interpretation [as in (1c)]. But example (2), with a perceptual verb (*ver* “to see”) is three-way ambiguous in Spanish: it has the same HA and LA interpretations available in (2) plus a third one if we interpret the clause as a PR. This interpretation is eventive, equivalent to the English sentence *I saw the son of the baker running in the park*. In languages like Spanish, this eventive structure is string identical to a relative clause—hence the term *Pseudorelative*.

Crucially, PRs can only attach high, that is, *the baker* cannot be seen running; it must always be the higher NP *el hijo* (the son). Grillo and Costa (2014) argued this is why some languages reveal HA preferences: because they have PRs. Pseudorelatives are only possible when the matrix verb is perceptual (e.g., “to see”, “to hear”, “to watch” etc.), and the embedded and matrix verbs must share tense, while the embedded verb has imperfective aspect (see Grillo and Costa, 2014 for further details on the grammar of pseudorelatives).

The PR First Hypothesis claims that in cases of ambiguity between a PR reading or a RC reading, all else being equal, the PR parsing takes priority. This is because, the authors claim, PRs are structurally and semantically simpler than RCs. Conversely, when a true RC is present—a string that cannot be interpreted as a PR—the parser will follow Late Closure and Minimal Attachment, thus favoring LA.

The predictions of the PR-First Hypothesis are hence the following:

i All sentences allowing a PR reading attach high.
ii Otherwise, all RCs attach low.

A number of recent studies have tested the PR-First Hypothesis in Portuguese (Costa et al., 2016; Tomaz et al., 2014), Spanish (Aguilar and Grillo, 2021; Aguilar et al., 2021, 2022; García and Sandoval, 2017), French (Pozniak et al., 2019) and Italian (Grillo and Costa, 2014; Grillo and Turco, 2016; Lee and De Santo, 2024), using either online or offline measures and have argued to have found support for it. However, these studies have significant limitations that we briefly discuss below.

For instance, let us take the result from an offline questionnaire in Italian reported by Grillo and Costa (2014). We argue that the results obtained do not meet their predictions. In questionnaire 1, the authors created four different conditions manipulating (i) the position of the embedded clause, either right-branching or center-embedding, and (ii) the site of extraction, resulting in either a subject or an object extraction. Only one of the four conditions, the right-branching subject-extracted one, admitted a PR reading. Participants were asked to provide their preferred attachment for 20 items allocated in a latin-square design. Results showed that the rates for HA in the PR-available condition were 56.6%, which is not what the Hypothesis predicts. Also, in the RC-only conditions, where only LA preference is predicted, they still obtained a rate of HA preference ranging between 32.8% and 44%. While they did find a significant difference between conditions, suggesting a stronger preference for HA in PR-available contexts compared to RC-only contexts, the categorical predictions of the PR-First Hypothesis are not met.

In other cases, such as in Aguilar and Grillo (2021), the attachment preferences are random, that is, the participants, overall, did not prefer either LA or HA. The authors were interested in understanding whether the aspectual properties of the embedded verb modulated attachment preferences. They created 24 experimental sentences with 4 different conditions, manipulating (i) the matrix verb, either perceptual or non-perceptual, and (ii) the aspect of the embedded verb, either imperfective or progressive. Only two out of the four conditions permitted a PR reading (the ones introduced by a perceptual verb). The authors reported a 51% preference for HA in one of the PR-available conditions in Experiment 1, and a 55.2% preference for high attachment in one of the PR-available conditions of Experiment 2.

Here, we present further evidence that the PR-First Hypothesis cannot account for HA preferences, by means of two self-paced reading experiments in Spanish (preregistered at https://osf.io/4gyt7). To this purpose, we carefully designed and tested the materials. We also ensured sufficient statistical power, controlled for the lexical frequency of each sentence element, the length of the materials, and the availability of the PR interpretation in the selected matrix verbs.

## 2 Experiment 1: testing the processing cost of high- vs. low-attachment

The PR-First Hypothesis agrees with Frazier (1979) that HA is costlier than LA. It also claims that PRs (obligatorily HA) are easier to process than RCs, so that a HA preference in Spanish must be due to PR readings (Grillo and Costa, 2014). The aim of Experiment 1 is to test these claims. In order to do it, we designed a self-paced reading experiment with non-ambiguous sentences in Spanish containing an embedded clause of the type [NP1 *de* NP2] [*que …*]. These materials were then manipulated to generate the materials for four experimental conditions. Either the matrix verb (MV) was perceptual and thus compatible with a PR reading for the embedded sentence, which should force HA, or it was not, forcing a RC interpretation where LA ought to be preferred. These experimental sentences forced attachment either on NP1 or NP2 by means of number agreement, as shown in (4), where the four conditions are presented:

(2)
  a. Perceptual matrix verb, LA:
  *Carmen vio al nieto de los comerciantes que fumaban delante del hospital*.
  Carmen saw the grandson of the shopkeepers that were smoking in front of the hospital.
  b. Perceptual matrix verb, HA (PR-available):
  *Carmen vio a los nietos del comerciante que fumaban delante del hospital*.
  Carmen saw the grandsons of the shopkeeper (that were) smoking in front of the hospital.
  c. Non-perceptual matrix verb, LA:
  *Carmen llamó al nieto de los comerciantes que fumaban delante del hospital*.
  Carmen called the grandson of the shopkeepers that were smoking in front of the hospital.
  d. Non-perceptual matrix verb, HA:
  *Carmen llamó a los nietos del comerciante que fumaban delante del hospital*.
  Carmen called the grandsons of the shopkeeper that were smoking in front of the hospital.

Before proceeding with the experiments, we conducted two norming studies: Norming Study 1 controlled for possible semantic biases toward high or low attachment, and Norming Study 2 ensured the availability of PRs with the previously selected perceptual matrix verbs.

### 2.1 Norming study 1

In order to control for potential semantic biases, and to ensure that both the NP1s and the NP2s of each experimental item related with similar naturalness to the embedded verb [i.e., that in (4), that both “a grandson” and “a shopkeeper” are equally likely to be smoking in front of the hospital], we conducted a web-based norming questionnaire on the Ibex Farm platform (Drummond, 2013) for the sentences created for Experiments 1 and 2. The norming study contained simple, non-embedded sentences with either the NP1 [as in (5a)] or NP2 [as in (5b)] as subjects of the verbs to be used in the embedded clauses in the experiment.

(5)
  a. NP1 *El nieto fumaba delante del hospital*.
  The grandson was smoking in front of the hospital.
  b. NP2 *El comerciante fumaba delante del hospital*.
  The shopkeeper was smoking in front of the hospital.

A linear model showed that the normalized frequency—as gathered in the CREA corpus by Real Academia Española (2008)—of the nouns selected as NP1s (mean = 71.25, SD = 70.66) and NP2s (mean = 77.23, SD = 83.87) did not differ [*F*_(1, 82)_ = 1.39, *p* = 0.24]. Moreover, Zipf values for all NPs were above 4, to avoid low-frequency words.

Participants (*N* = 120, 80 females, mean age = 35.758; SD = 12.933, all of them natives of peninsular Spanish) undertook the questionnaire on Ibex Farm, remotely and at their own pace. They were asked to read each sentence carefully and evaluate their naturalness and acceptability on a 7-point Likert-scale, being 1 = *totally unacceptable* and 7 = *totally acceptable*. No feedback was offered. We compared the naturalness of the two conditions and excluded those items in which there was a difference between the two conditions, in order to ensure that the items used in Experiments 1 and 2 have no semantic bias. Trials in which a participant answered faster than 1,000 ms were discarded (< 0.001% of the data).

The data were analyzed by using R Studio software (R Core Team, 2020). Both the median and the mode for highly-unacceptable filler items were 1. For highly-acceptable fillers, the median and the mode were 7. The median for each experimental item individually was 7, as well as the mode. As for the experimental items, the median was 7 for the overall sentences, meaning that the experimental items were highly natural. Furthermore, the median was also 7 for each experimental item individually and for each condition. Then, we tested the overall ratings against chance level. A one-tailed one-sample Wilcoxon test against μ = 4 revealed a significant difference (*V* = 5,459,664, *p* < 0.001), suggesting that the data did differ from chance level. Finally, we tested the ratings of one condition against the other. A Mann-Whitney-Wilcoxon test for independent samples did not reveal any difference (*W* = 804137, *p* = 0.316).

Results showed overall high scores, above chance level, indicating that the items were well-formed and highly natural. Furthermore, the scores from the two conditions did not differ. We concluded that the NPs of the items created for Experiment 1 were equally plausible to carry on the action of the embedded verb, given that there was no difference between the two conditions.

### 2.2 Norming study 2

We conducted a second norming study, a web-based questionnaire on the Ibex Farm platform (Drummond, 2013), in order to assess whether speakers indeed allowed PR readings with perceptual matrix verbs in Spanish. Each item started with a matrix perceptual verb followed by an object NP and a clause headed by *que* “that”, as shown in (6), with obligatory PR reading, as proper nouns do not allow RCs.

(6) *Vi a Marta que patinaba con sus amigas*.
I saw Marta skating with her friends.

The normalized frequencies of the perceptual matrix verbs (MVs) ranged greatly, from < 1 occurrence per million to over a thousand occurrences per million. This allowed testing for the broadest possible number of verbs.

We then created 21 ungrammatical filler sentences, and 21 highly acceptable and natural filler sentences. Each participant saw half of the experimental sentences (*N* = 14, thus resulting in the creation of two lists) and all the fillers. The whole experiment lasted around 5 min. The procedure was the same as in Norming Study 1.

We discarded data from those participants whose answers on the ungrammatical fillers differed the most from the expected outcome (i.e., 1 = “totally unacceptable”). As a result of this trimming process, data from 60 participants (29 females, mean age = 39.05; SD = 10.682) were analyzed. Furthermore, all the trials in which a participant answered faster than 1,000 ms were discarded (< 0.001% of the data).

The data were analyzed by using R Studio software (R Core Team, 2020). Both the median and the mode for highly-unacceptable filler items were 1. For highly-acceptable fillers, the median and the mode were 7. We checked the overall median and mode for the experimental sentences, to ensure the availability of PRs. The median was 5, the mode was 7. Furthermore, we tested the ratings of the experimental ratings against chance (μ = 4). A one-tailed one-sample Wilcoxon test against μ = 4 revealed a significant difference (*V* = 169,076, *p* < 0.001). Of the experimental items, 16 had median value higher than 4.

Given the high values of mode and median, we concluded that PRs are available and acceptable for native speakers of peninsular Spanish. We also concluded that at least 16 of the perceptual verbs could introduce PRs, i.e., those which received median score >4, and, consequently, we used those verbs to craft the materials for Experiment 1.

Subsequently, we selected 10 of the highest-rated MVs to use in Experiment 1, and matched them with other 10 non-perceptual verbs to create the conditions in (4)c and (4)d. All of these verbs had Zipf values >4. A Wilcoxon test showed that the normalized frequency—as gathered in the CREA corpus by Real Academia Española (2008)—of perceptual verbs (mean = 261.7, SD = 457.51) and of non-perceptual verbs (mean = 217.31, SD = 263.82) did not differ (*W* = 51, *p* = 0.97). Again, Zipf values for all non-perceptual verbs were above 4, to avoid words of low frequency.

These two Norming studies ensured that the materials created for the experiments were natural and free of biases that might modulate attachment preferences. By doing so, we ensured that the results obtained in Experiments 1 and 2 are unlikely to be due to flaws in the materials and/or to an imbalance in features between conditions, such as differences in word frequency and/or plausibility between the two attachments.

### 2.3 Participants

In order to ensure a large pool of participants, half of them were tested in-lab and half of them in an identical online version. A total of 161 participants took part in Experiment 1 (81 in-lab, 54 females, mean age = 24.25, SD = 7.58; 80 internet-based, 67 females, 1 did not want to disclose this information, mean age = 24.52, SD = 6.69). Due to an error in the distribution of the lists, data of one participant were removed from the analysis and an additional participant was tested to preserve the balance in the number of observations per list. All participants were offered compensation for their time and gave their informed consent under experimental protocols approved by the Ethics Committee of the UPV/EHU (*Comité de Ética para las Investigaciones relacionadas con Seres Humanos*, CEISH: M10_2020_182). All participants were native speakers of peninsular Spanish. Their dominant language was Spanish, that is, they mainly spoke Spanish with their families and acquaintances, and carried out their daily activities and jobs almost exclusively in Spanish, as assessed via a questionnaire.

### 2.4 Materials

We selected 20 NPs as NP1s, and another set of 20 NPs as NP2s. We matched NP1s and NP2s appropriately and generated 40 combinations of complex NPs of the [NP1 of NP2] type. We assigned each of the NPs to a pair of perceptual and non-perceptual matrix verbs, resulting in 40 non-ambiguous sentences like the ones shown in (4). For each item, we created four conditions manipulating (i) Attachment, forcing it to be either HA or LA, and (ii) the Matrix Verb, making it perceptual or non-perceptual. In the HA condition, the embedded verb agreed with NP1, while in the LA condition the verb agreed with NP2.

### 2.5 Procedure

The experiment was prepared and initially run on the Ibex Farm platform (Drummond, 2013), and, after its demise, on the updated version PCibex (Zehr and Schwarz, 2018). Participants took the experiment in the Experimental Linguistics Laboratory at the Micaela Portilla Research Center of the University of the Basque Country in Vitoria-Gasteiz, having been instructed to proceed at their own pace, or they performed the task online.

Participants filled a survey form, provided demographic and linguistic information, and were instructed as to how to carry on the experiment. They were presented at first with a blank screen with underscores placed where the words of the stimuli would appear. They were asked to press the space bar to read the sentences word-by-word at their own pace (self-paced reading method). Whenever a new word appeared on the screen, the previous one would disappear. All stimuli were presented on the same line, to avoid breaks in their implicit prosody that could bias attachment (Fodor, 2002; Hemforth et al., 2015). At the end of each sentence, a comprehension question appeared in the screen. In the case of experimental items, the question inquired about who was carrying out the activity on the embedded verb. The NP1 and NP2 would appear on the screen as answers for this comprehension question, and participants had to select their answer on the keyboard by using the key “A” for the left-hand answer, and “L” for the right-hand answer. The position of the answers was random, so to have a 50% of correct answers on the left side, and vice versa. There was no time limit on this task. No feedback was offered. The first six sentences were practice trials to ensure familiarization with the task. Participants were not informed about the practice trials, and no feedback was offered. Practice trials were excluded from the analyses. The presentation of the stimuli was pseudo-randomized: three filler sentences were randomly placed between each two experimental items (which were also randomized).

### 2.6 Data preparation

The data were analyzed using R Studio software (R Core Team, 2020) and the lme4 (Bates et al., 2015), afex (Singmann et al., 2021) and emmeans (Lenth, 2022) packages.

First, participants aware of experimental manipulations used in the study were discarded (*N* = 3). Regarding the in-lab modality, one participant was excluded because she reported having read the sentences out loud. After such a trimming, the final pool consisted of 76 participants for the in-lab modality (51 females, mean age = 22.75; SD = 7), and 77 participants for internet modality (data of 3 participants were excluded from the analysis due to an accuracy score lower than 70%: 64 females, 1 did not want to disclose this information, mean age = 24.67, SD = 6.76). Data from a total of 153 participants were analyzed.

We subsequently calculated the logarithmic reading times for each word. Then, following the steps described in Jaeger's blog (Jaeger, 2007, 2008), we calculated the residual reading times for the critical and post-critical regions, separately for each experiment modality—laboratory or internet. In other words, we ran a linear mixed model on the logarithmic reading times, with item ID, the word length, the logarithmic position of the stimulus in the list, the position of the word in the sentence as fixed effects; and subject ID as random effect.

We first excluded from the analysis the data from participants who had scored < 70% in accuracy. Subsequently, we trimmed all those items in which a participant had read any of the words in fewer than 50 ms or in more than 3,000 ms, which resulted in the deletion of 5.4% of in-lab participants, and 4.63% of those who performed the experiment at home. Next, we deleted all reading times that exceeded a threshold of 2.5 standard deviations from the mean by participant, region and condition. We also removed all response times and corresponding answers that exceeded the same threshold, which resulted in an overall deletion of the 8.23% of the data in the laboratory sample, and of 7.35% in the internet sample. Finally, all response times and corresponding answers lower than 500 ms or higher than 7,000 ms were discarded, as well. Over all, we removed the 8.54% of the data from the in-lab part of the experiment and 7.57% of the internet version of the experiment.

### 2.7 Data analysis

Accuracy was analyzed using a generalized linear mixed model on binomial data. The best-fitting model was selected by means of an ANOVA (analysis of variance) between the full model and a series of simplified models, selecting the simplest and best-fitting model. The full model included Matrix Verb (perceptual vs. non-perceptual), Attachment (high vs. low) and their interaction as predictors; and participant code, item number and modality (laboratory vs. internet) as random effects. The best-fitting model was a model with Attachment as predictor.

Response times were analyzed using a linear mixed model on the logarithmic times. The best-fitting model was selected by means of an ANOVA (analysis of variance) between the full model and a series of simplified models, selecting the simplest and best-fitting model. The full model included Matrix Verb (perceptual vs. non-perceptual), Attachment (high vs. low) and their interaction as predictors; and participant code, item number and modality (laboratory vs. internet) as random effects. The best-fitting model was a model with Matrix Verb as predictor. Only correctly answered trials were analyzed.

Reading times at all regions of interest (Embedded Verb and the following two regions: EV, EV+1, EV+2) were analyzed using a linear mixed model on the residual reading times. The best-fitting model was selected by means of an ANOVA (analysis of variance) between the full model and a series of simplified models, selecting the simplest and best-fitting model. The full model included Matrix Verb (perceptual vs. non-perceptual), Attachment (high vs. low), their interaction as predictors, as well as the logarithmic reading times from the two previous regions—which were never excluded in any of the models and were not taken into account when significant; and participant code, item number and modality (laboratory vs. internet) as random effects. The best-fitting model at the embedded verb was a model with Attachment as predictor; and a model with no predictor in the following two regions. Only correctly answered trials were analyzed.

### 2.8 Predictions

Taking into account the example item in (4), the PR-First Hypothesis predicts condition (b)—that is, a high-attaching sentence introduced by a perceptual verb, allowing a PR reading—to be the easiest to process, and condition (d)—a high-attaching structure, introduced by a non-perceptual verb, and only allowing an RC reading—to be the most costly. Participants were consequently expected to perform the task faster and more accurately on condition (b) than on the other conditions. Also, participants should be slowest and least accurate in condition (d). Similarly, the time taken to answer the comprehension questions should be faster in condition (b) than in the other ones, particularly than in condition (d), which should take them longest. The same should be true for reading times at the critical and/or post-critical regions.

## 3 Experiment 1: results

**Figure 2:**
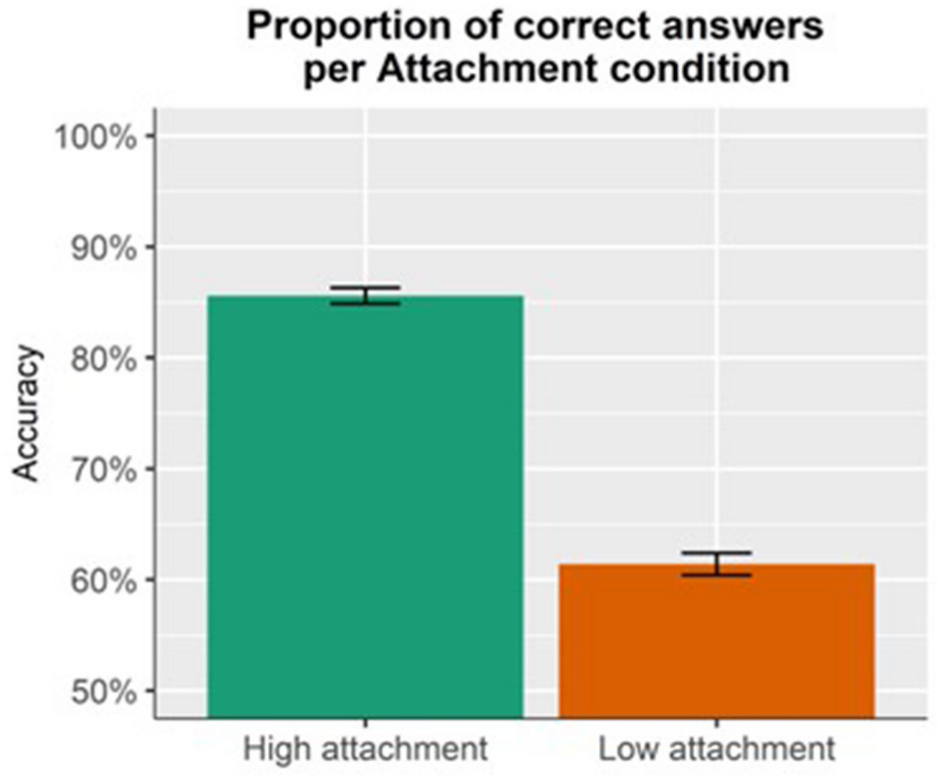
Proportion of correct answers per each attachment condition. High-attaching sentences were more accurate than low-attaching sentences.

Results for accuracy (Figure 2) showed a main effect of Attachment (*p* < 0.001), indicating that responses to high-attaching sentences (mean = 0.86, SD = 0.35) were more accurate than to low-attaching sentences, regardless of PR availability (mean = 0.61, SD = 0.49).

**Figure 3:**
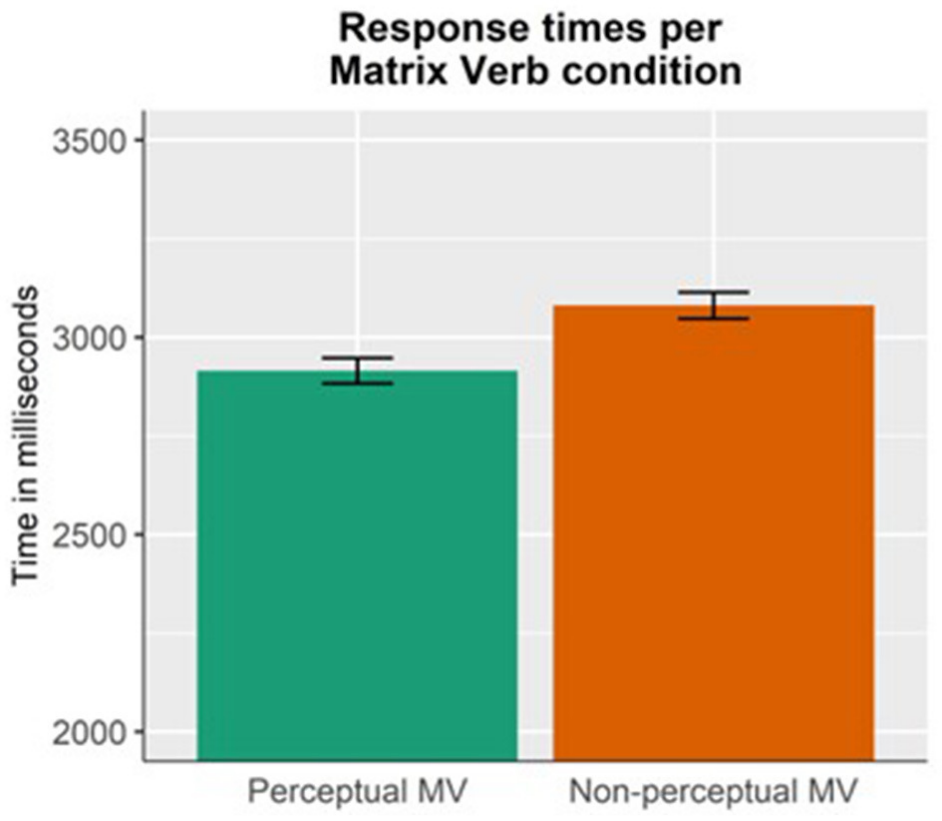
Response times per each matrix verb condition (perceptual vs. non-perceptual MV). Answers to the sentences introduced by perceptual matrix verbs were faster than those introduced by non-perceptual matrix verbs, regardless of the manipulation of Attachment.

Results for the response times (Figure 3) showed a main effect of Matrix Verb (*p* = 0.01), that is, the questions to sentences introduced by a perceptual matrix verb (mean = 2,915 ms, SD = 1,376 ms) were answered faster than the questions introduced by a non-perceptual matrix verb (mean = 3,080 ms, SD = 1,424 ms).

Results for the reading times at the embedded verb revealed a main effect of Attachment (*p* = 0.04): verbs in high-attaching sentences (mean = 474.89 ms, SD = 245.72 ms) were faster to read than those in low-attaching sentences (mean = 481.11 ms, SD = 265.93 ms). Results for the reading times at the post-critical regions showed no effect or interaction for any of the predictors into consideration.

In sum, the results of the Experiment 1 revealed a general preference for high attachment, but this preference was not modulated by the PR availability.

## 4 Experiment 2: materials and methods

Experiment 1 showed that HA is faster and easier to process in Spanish, regardless of PR availability. That is, PR readings do not appear to be less costly than HA-RC readings, contrary to the claims in Grillo and Costa (2014) about the structural simplicity and processing ease of Pseudorelative readings. Next, we conducted an additional self-paced reading experiment with ambiguous sentences, designed to test the PR-First Hypothesis directly (preregistered at https://osf.io/ph72t). This experiment allowed us to gather information about the preferred attachment strategy by asking the participants whether they interpreted each experimental sentences as high- or low-attaching. Furthermore, we measured the cost of attachment choices by means of reading and response times. In other words, we cross-analyzed the online results with the attachment preferences reported by the participants.

The PR-First Hypothesis predicts that in PR-available environments there will be high-attachment preferences, while in RC-only environments there will be low-attachment preferences. It also predicts faster reading and response times whenever a Pseudorelative reading is possible. Conversely, in the RC-only condition, the hypothesis predicts faster reading and response times when low attachment is selected by participants.

The materials were the same as in Experiment 1, therefore, grammatical and/or lexical biases were prevented by means of two norming studies.

### 4.1 Participants

All in all, 80 participants took part in Experiment 2 (40 in-lab, 27 females, mean age = 22.97, SD = 6.35; 40 internet-based, 31 females, mean age = 26.25, SD = 4.04). Some of the participants preferred not to be paid, but all participants were offered compensation for their time and gave their informed consent under experimental protocols approved by the Ethics Committee of the UPV/EHU (*Comité de Ética para las Investigaciones relacionadas con Seres Humanos, CEISH:* M10_2020_182). All participants were native speakers of peninsular Spanish. Their dominant language was Spanish, as assessed via a questionnaire.

### 4.2 Materials

Materials for this experiment were created from the materials in Experiment 1, and were turned ambiguous by making NP1s, NP2s and EVs singular. Materials were normed as detailed above for Experiment 1 (see an example in 7). The items were either introduced by a perceptual MV thus allowing a PR reading as in (7a), or by a non-perceptual MV thus forcing a RC reading as in (7b).

(7)
  a. Perceptual matrix verb (PR-available):
  *Carmen vio al nieto del comerciante que fumaba delante del hospital*.
  Carmen saw the grandson of the shopkeeper (that was) smoking in front of the hospital.
  b. Non-perceptual matrix verb (RC-only):
  *Carmen llamó al nieto del comerciante que fumaba delante del hospital*.
  Carmen called the grandson of the shopkeeper that was smoking in front of the hospital.

### 4.3 Procedure

The procedure was the same as in Experiment 1.

### 4.4 Data preparation

The data were analyzed by using R Studio software (R Core Team, 2020) and the lme4 (Bates et al., 2015), afex (Singmann et al., 2021) and emmeans (Lenth, 2022) packages.

We first calculated the residual reading times separately per each modality, as detailed for Experiment 1. The final pool consisted of 78 participants (in-lab: 27 females, mean age = 22.92, SD = 6.42; internet modality: 27 females, mean age = 27.82, SD = 8.3). Data of two participants were excluded from the analyses due to diagnosed dyslexia (one participant in-lab) and to a low accuracy score (one participant internet modality). To clean the data, we discarded those participants who scored < 70% of accuracy in the filler items. To clean the data, we discarded those participants who scored < 70% of accuracy in the filler items. One participant (internet modality) was discarded in this step. All items in which a participant had read any of the words in < 50 ms or more than 3,000 ms were trimmed, resulting in the overall deletion of 14.3% of the data. All reading times that exceeded a threshold of 2.5 standard deviations from the mean by participant, region and condition were deleted. All response times (and their corresponding answers) that exceeded the same threshold (overall deletion of 13.8% of the data) were deleted as well. Finally, all response times (and corresponding answers) lower than 500 ms or higher than 7,000 ms were also discarded (overall, 17.96% of the data).

### 4.5 Data analysis

Attachment preferences were analyzed using a generalized linear mixed model on binomial data. The best-fitting model was selected by means of an ANOVA (analysis of variance) between the full model and a series of simplified models, selecting the simplest and best-fitting model. The full model included Matrix Verb (perceptual vs. non-perceptual) as predictor, and participant code, item number and modality (laboratory vs. internet) as random effects. The best-fitting was one with no predictors.

Response times were analyzed using a linear mixed model on the logarithmic times. The best-fitting model was selected by means of an ANOVA (analysis of variance) between the full model and a series of simplified models, selecting the simplest and best-fitting model. The full model included Matrix Verb (perceptual vs. non-perceptual), Attachment preference (high vs. low), and their interaction as predictors; and participant code, item number and modality (laboratory vs. internet) as random effects. The best-fitting model included Attachment preference as predictor. Only correctly answered trials were analyzed.

Reading times at all regions of interest (the Embedded Verb and the following two regions: EV, EV+1, EV+2) were analyzed using a linear mixed model on the residual reading times. The best-fitting model was selected by means of an ANOVA (analysis of variance) between the full model and a series of simplified models, selecting the simplest and best-fitting model. The full model included Matrix Verb (perceptual vs. non-perceptual), Attachment preferences (high vs. low) and their interaction as predictors; and participant code, item number and modality (laboratory vs. internet) as random effects. The best-fitting model for the reading times at the first two critical regions (embedded verb and following region: EV and EV+1) was a model with no predictors. The best-fitting model at the last critical region was a model with Attachment preference as predictor. Only correctly answered trials were analyzed.

### 4.6 Predictions

Taking into account the example item in (7), the PR-First Hypothesis (Grillo and Costa, 2014) predicts that condition (7a), which is ambiguous between a PR and a RC reading, should yield high attachment exclusively; whereas condition (7b), in which only RC is available, would receive a preference for low attachment exclusively—in line with Minimal Attachment and Late Closure (Frazier, 1979). This is so because (7a) admits PRs, and, therefore, according to the PR-First Hypothesis, such interpretation will be preferred, whereas (7b) only admits RCs. The PR-First Hypothesis also predicts that, whenever high attachment is preferred in (7a), reading and response times will be faster when compared to a low attachment preference in the same condition. Conversely, in (7b), reading and response times associated to a low attachment preference would be faster than those associated to a high attachment preference in the same condition.

## 5 Results

**Figure 4:**
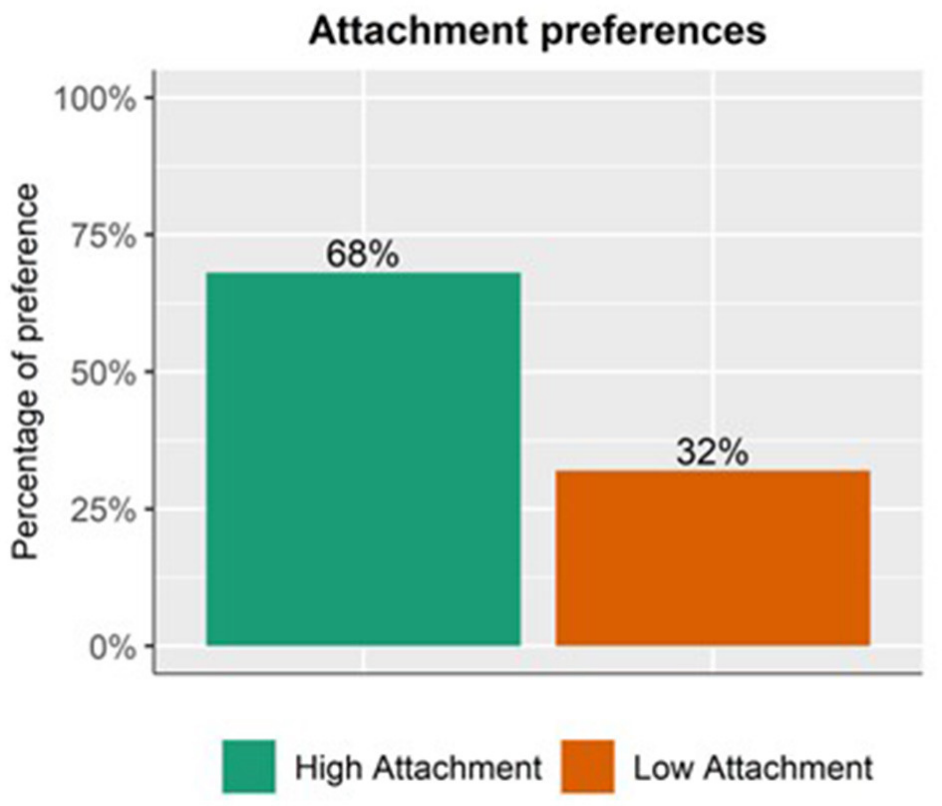
Proportion of overall preferences. High attachment was preferred more often than low attachment.

The intercept of the model was significant (*p* < 0.001). This indicates that high-attachment preference was higher than chance (50%). No main effect of Matrix verb was found (Figure 4).

**Figure 5:**
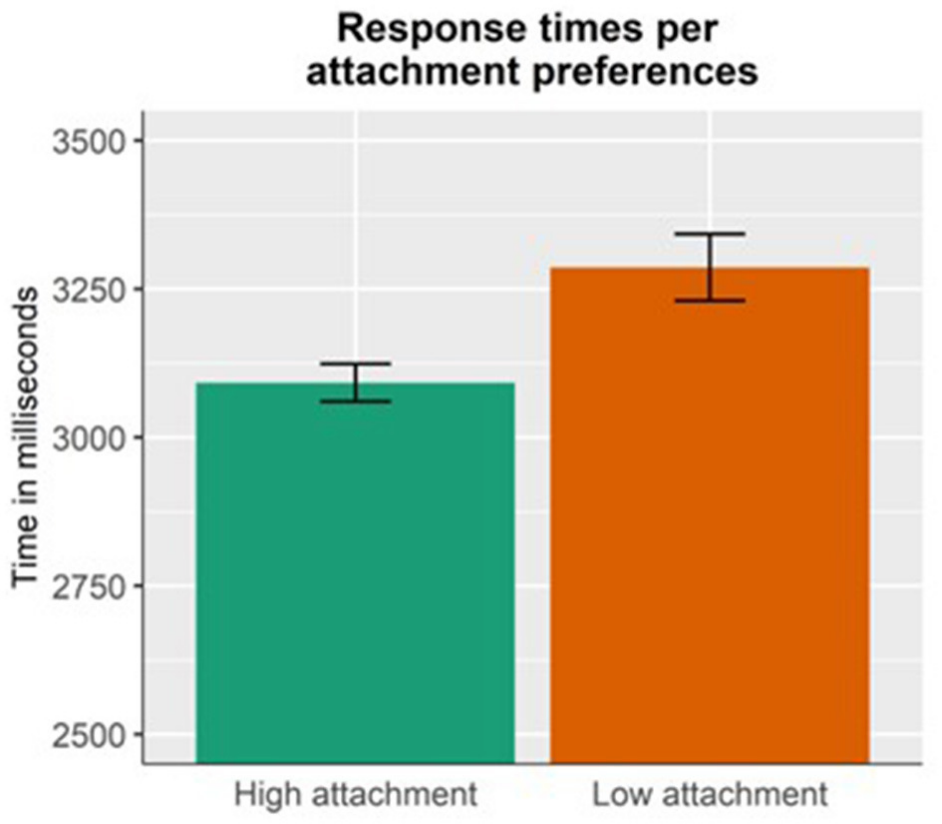
Response times per each attachment preference (high or low attachment). HA choices were answered faster than LA choices.

Results for the response times (Figure 5) show a main effect of Attachment preference (*p* = 0.03), indicating that participants were faster when making high-attachment choices (mean = 3,092 ms, SD = 1,283 ms), as compared to low-attachment ones (mean = 3,286 ms, SD = 1,553 ms).

Results for the reading times at the critical (EV) and immediate post-critical region (EV+1) showed no effect or interaction. As for the last critical region (EV+2), there was a main effect of Attachment preference (*p* = 0.02) showing that, at this last critical region, HA sentences (mean = 397.62 ms, SD = 161.96 ms) were read faster than LA ones (mean = 400.25 ms, SD = 176.58 ms).

In sum, the results of the Experiment 2 revealed that participants preferred to interpret ambiguous sentences as HA regardless of PR availability, contrary to what the hypothesis predicts.

## 6 Discussion

In this paper, we presented the results of two experiments testing the PR-First Hypothesis. In Experiment 1, we used the self-paced reading method to compare the processing cost of high vs. low attachment in non-ambiguous sentences in Spanish. To our knowledge, no experimental evidence has ever been gathered to directly test Frazier's claim about the cognitive cost of high attachment with non-ambiguous materials in Spanish. The use of unambiguous items, whose attachment was forced to be either high or low, allowed us to test which option was easier to process. Results showed higher accuracy and faster reading times for HA than for LA, regardless of PR availability. Similarly, our results showed a clear advantage for high attachment, regardless of the type of matrix verb (perceptual or not), in both offline and online measures. According to Frazier's *Minimal Attachment* and *Late Closure* principles, high attachment should be the costliest construction to process, but our evidence showed that it is the easiest parsing choice.

Results from Experiment 1 did not support the PR-First Hypothesis either. This Hypothesis predicts that high attachment preference is due to the availability of PRs, which are easier to parse than RCs. We found a consistent advantage of high attachment in all contexts, not only when a pseudorelative clause was available.

Finally, we found an advantage for perceptual matrix verbs in the response times, that is, the questions to sentences introduced by perceptual matrix verbs were answered faster than the questions introduced by non-perceptual matrix verbs. This result cannot be due to a higher frequency of the perceptual matrix verbs over the non-perceptual ones, because this factor had been controlled (see the Norming Studies 1 and 2). It is not because a perceptual matrix verb prototypically introduces a pseudorelative, which could make sentences with perceptual verbs easier to parse, as only 50% of the items with such verbs allow a pseudorelative reading. In other words, half of the experimental materials introduced by perceptual matrix verbs were followed by high-attachment RC, i.e., “Carmen saw the grandsons of the shopkeeper (that were) smoking (…)”, while the other half were followed by low-attachment RC, i.e., “Carmen saw the grandson of the shopkeepers (that were) smoking (…)”. We cannot draw any clear conclusion on the issue because the experiments were not designed to test this hypothesis. Therefore, further research will determine the underlying reasons behind the pattern of results observed in our experiments.

Our findings are consistent with most of the literature since Cuetos and Mitchell (1988) pioneer work and up to Grillo and Costa (2014). The present results showed that Spanish participants experience an advantage for high-attaching sentences with complex NPs, not only when PRs are available, but across-the-board.

Regarding the findings from Experiment 2, data revealed more high attachment readings of the ambiguous sentences than low attachment readings overall, regardless of the condition, thus indicating a strong high-attachment preference. Results also indicated shorter reading and response times for high attachment interpretations as compared to low attachment ones. Similar to Experiment 1, results revealed a preference and facilitation for high attachment over low attachment, with no modulation depending on the matrix verb (attachment preferences, response times and reading times at the last critical region EV+2). Such results did not support Late Closure, Minimal Attachment, or the PR-First Hypothesis; instead, they were in line with the original results reported in Cuetos and Mitchell (1988).

Furthermore, unlike Baccino et al. (2000), De Vincenzi and Job (1995), and Kamide and Mitchell (1997) who found differences between offline attachment preferences and online data regarding the processing cost of either RC attachment, we did not find such a difference in our work.

Taken together, our results showed a consistent preference and facilitation for high attachment in Spanish, both in offline and online measures. Rohde et al. (2011) provided evidence for increased high-attachment preferences when the main verb could trigger implicit causality. Some of our RC-only experimental items from Experiment 2 included sentences such as *Alberto envidió al colega del gobernador que sal*í*a con mi prima* (“Albert envied the colleague of the governor who left with/dated my cousin”). The use of the verb *envidiar* (“to envy”), following Rohde et al. (2011) could have biased the participants' preference for high attachment, since they could have read the item as “Albert envied the colleague of the governor *because* he left with/dated my cousin”. However, since our materials included only three verbs that could have triggered implicit causality (*ayudar* “to help”, *regañar* “to scold”, and *envidiar* “to envy”), we believe the general preference for high attachment is unlikely to be attributed to this factor. Future studies should consider this limitation when designing their materials.

Considering all the evidence, our results showed no support for the PR-First Hypothesis. High attachment was overall preferred and facilitated, and PR availability did not modulate the results. PR-First predicts that results would be determined by PR availability (Grillo and Costa, 2014) and no high attachment preference would be found in RC-only contexts. Our results did not support these predictions.

In line with Alonso-Pascua (2020), we argue that the PR-First Hypothesis is not supported by the results from the previous works. The reason is that the hypothesis predicts almost complete preference for high attachment in PR-available contexts in all languages admitting PRs; and almost no preference or facilitation for high-attaching relative clauses. However, none of the works published so far found an absence of high-attachment preference for relative clauses.

Furthermore, we suggest that the hypothesis only applies to a reduced type of constructions, and does not account for structures that allow, for instance, implicit causality, or other modulating factors, such as animacy and RC length. In short, it applies to a small group of instances, and it can hardly generalize to offer the key to understanding how relative clause attachment works in human minds.

Instead, we suggest that PR availability could be one of the many modulating factors for attachment preferences in *some languages*, along with animacy (Hsiao and MacDonald, 2016; Kwon et al., 2019), prosody (de la Cruz-Pavía and Elordieta, 2015; Fernández and Sekerina, 2015; Hemforth et al., 2015; Mahmoodi et al., 2022) and linguistic profile (de la Cruz-Pavía and Elordieta, 2015; Jegerski et al., 2016a,b; Mahmoodi et al., 2022; Marefat et al., 2015; Marefat and Farzizadeh, 2018) among others. According to this view, PR availability does not determine attachment preferences, but it could modulate them toward high attachment, and, therefore, future works should take this effect into account. There is evidence that the variation in attachment preferences is a multifactorial issue, which cannot be accounted for by means of a single, categorical factor such as PR availability, as claimed in the PR-First Hypothesis, or locality, as claimed by Frazier (1979).

Furthermore, it would be advisable for future works to pre-test experimental materials to obtain similar plausibility between NP1- and NP2-attaching embedded verbs, as most of the aforementioned works did (Norming Study 1), and to pre-test the pool of perceptual verbs to be used in further experiments for their plausibility to introduce a PR (Norming Study 2).

In conclusion, in the present work, we tested high attachment preferences in Spanish and examined whether this preference could be explained by the PR-First Hypothesis. Our findings indicated that the PR-First Hypothesis does not explain attachment preferences and further studies will need to clarify the reason(s) why there is a crosslinguistic difference in attachment preferences.

## Data Availability

The raw data supporting the conclusions of this article will be made available by the authors, without undue reservation.
