# Supplementary material for: The actress was not on the balcony: testing the Pseudorelative-First Hypothesis in Spanish
Source: Front Psychol. 2025 Mar 5;16:1546432. doi: 10.3389/fpsyg.2025.1546432 (PMC11920142; doi:10.3389/fpsyg.2025.1546432)
Supplement: Supplementary file 1 [file Supplementary_file_1.docx]

Supplementary Material

# Supplementary Tables

**Descriptives of the different conditions for Experiment 1**

Accuracy

- Best-fitting model predictor(s): Attachment
- Intercept: estimate = 2.31, SE = 0.13, z = 17.16, p < 0.001
- Low Attachment: estimate = -1.61, SE = 0.12, z = -13.46, p < 0.001

| Accuracy in % | Perceptual MV | Non-perceptual MV | Total |
| --- | --- | --- | --- |
| High attachment | 83.5% | 87% | 85.3% |
| Low attachment | 60.4% | 62.3% | 61.3% |
| Total | 73.9% | 72.9% | 100% |

Response times

- Best-fitting model predictor(s): Matrix Verb
- Intercept: estimate = 7.95, SE = 0.04, t = 197.87, p < 0.001
- Perceptual MV: estimate = -0.05, SE = 0.02, t = -2.52, p = 0.01

| Mean and SD response times | Perceptual MV | Non-perceptual MV | Total |
| --- | --- | --- | --- |
| High attachment | 2856.9 ± 1300.14 | 3056.2 ± 1389.29 | 2953.25 ± 1347.35 |
| Low attachment | 3002.41 ± 1479.92 | 3113.18 ± 1469.51 | 3058.71 ± 1475.19 |
| Total | 2915.25 ± 1376.51 | 3080.42 ± 1423.82 | 2996.79 ± 1402.31 |

Reading times EV

- Best-fitting model predictor(s): Attachment
- Intercept: estimate = -1.22, SE 0.09, t = -13.53, p < 0.001
- Low Attachment: estimate = -0.02, SE = 0.01, t = -2.07, p = 0.0389

| Mean and SD reading times at EV | Perceptual MV | Non-perceptual MV | Total |
| --- | --- | --- | --- |
| High attachment | 476.02 ± 252.15 | 473.71 ± 238.92 | 474.89 ± 245.72 |
| Low attachment | 479.66 ± 271.46 | 482.54 ± 260.52 | 481.11 ± 265.93 |
| Total | 477.57 ± 260.47 | 477.59 ± 248.63 | 477.58 ± 254.64 |

Reading times EV+1

- Best-fitting model predictor(s): Null model
- Intercept: estimate = -0.03, SE = 0.08, t = -0.34, p = 0.73

| Mean and SD reading times at EV+1 | Perceptual MV | Non-perceptual MV | Total |
| --- | --- | --- | --- |
| High attachment | 442.13 ± 206.89 | 437.97 ± 199.33 | 440.09 ± 203.18 |
| Low attachment | 431.03 ± 173.48 | 444.72 ± 183.69 | 437.89 ± 178.75 |
| Total | 437.39 ± 193.35 | 440.92 ± 192.63 | 439.14 ± 192.98 |

Reading times EV+2

- Best-fitting model predictor(s):
- Intercept: estimate = -0.33, SE = 0.09, t = -3.81, p < 0.001

| Mean and SD reading times at EV+2 | Perceptual MV | Non-perceptual MV | Total |
| --- | --- | --- | --- |
| High attachment | 401.68 ± 148.22 | 403.61 ± 153.25 | 402.63 ± 150.69 |
| Low attachment | 417.99 ± 185.02 | 411.71 ± 166.33 | 414.85 ±175.88 |
| Total | 408.63 ± 165.07 | 407.14 ± 159.09 | 407.89 ± 162.11 |

# Supplementary Tables

**Descriptives of the different conditions for Experiment 2**

Attachment preferences

- Best-fitting model predictor(s): Null model
- Intercept: Estimate = 0.997, SE = 0.27, z = 3.65, p < 0.001

| Percentage (count / total items) | Perceptual MV | Non-perceptual MV | Total |
| --- | --- | --- | --- |
| High attachment preferences | 69% (842/1212) | 66% (791/1187) | 68% (1633/2399) |

Response times

- Best-fitting model predictor(s): Answer (attachment preference)
- Intercept: Estimate = 8.007, SD = 0.034, t = 237.703, p < 0.001
- High Attachment preference: estimate = -0.049, SE = 0.022, t = -2.186, p = 0.0297

| Mean and SD response times | Perceptual MV | Non-perceptual MV | Total |
| --- | --- | --- | --- |
| High attachment | 3104.59 ± 1332.39 | 3078.8 ± 1228.1 | 3092.09 ± 1282.61 |
| Low attachment | 3260.06 ± 1561.89 | 3311.1 ± 1546.69 | 3286.45 ± 1553.25 |
| Total | 3152.05 ± 1407.6 | 3156.3 ± 1346.62 | 3154.15 ± 1377.48 |

Reading times EV

- Best-fitting model predictor(s): Null model
- Intercept: estimate = -0.106, SE = 0.027, t = -3.957, p = 0.056

| Mean and SD reading times at EV | Perceptual MV | Non-perceptual MV | Total |
| --- | --- | --- | --- |
| High attachment | 462.16 ± 216.87 | 458.97 ± 221.44 | 460.6 ± 219.07 |
| Low attachment | 430.2 ± 205.82 | 459.77 ± 237.16 | 445.43 ± 222.89 |
| Total | 451.76 ± 213.78 | 459.24 ± 226.94 | 455.49 ± 220.44 |

Reading times EV+1

- Best-fitting model predictor(s): Null model
- Intercept: estimate = -0.083, SE = 0.013, t = -6.584, p < 0.001

| Mean and SD reading times at EV | Perceptual MV | Non-perceptual MV | Total |
| --- | --- | --- | --- |
| High attachment | 420.13 ± 175.65 | 420.8 ± 175.99 | 420.46 ± 175.77 |
| Low attachment | 416.81 ± 194.64 | 402.27 ± 145.86 | 409.32 ± 171.31 |
| Total | 419.06 ± 181.94 | 414.39 ± 166.37 | 416.73 ± 174.34 |

Reading times EV+2

- Best-fitting model predictor(s): Answer (attachment preference)
- Intercept: estimate = -0.07, SE = 0.054, t = 2.073, p = 0.323
- Low Attachment preference: estimate = 0.038, SE = 0.016, t = 2.363, p = 0.0185

| Mean and SD reading times at EV | Perceptual MV | Non-perceptual MV | Total |
| --- | --- | --- | --- |
| High attachment | 398.04 ± 165.09 | 397.19 ± 158.74 | 397.62 ± 161.97 |
| Low attachment | 405.68 ± 180.58 | 395.15 ± 172.78 | 400.25 ± 176.58 |
| Total | 400.5 ± 170.2 | 396.49 ± 163.66 | 398.5 ± 166.95 |

# Supplementary Data

**Supplementary materials for Norming Study 1: experimental sentences**

| List | Item | Condition | Stimulus |
| --- | --- | --- | --- |
| 1 | 1 | NP1-attaching | El hijo cantaba en el coro. |
| 1 | 2 | NP1-attaching | El compañero silbaba como un pastor. |
| 1 | 3 | NP1-attaching | El jefe tosía por el asma. |
| 1 | 4 | NP1-attaching | El niño estudiaba en la biblioteca. |
| 1 | 5 | NP1-attaching | El colega salía con mi prima. |
| 1 | 6 | NP1-attaching | El médico jugaba a fútbol sala. |
| 1 | 7 | NP1-attaching | El niño corría en el parque. |
| 1 | 8 | NP1-attaching | El maestro cocinaba en la cafetería. |
| 1 | 9 | NP1-attaching | El jefe comía en el restaurante. |
| 1 | 10 | NP1-attaching | El secretario conducía un viejo Seiscientos. |
| 1 | 11 | NP1-attaching | El sastre caminaba con unas muletas. |
| 1 | 12 | NP1-attaching | El suegro paseaba por el río. |
| 1 | 13 | NP1-attaching | El hermano robaba chicles del estanco. |
| 1 | 14 | NP1-attaching | El compañero bailaba danzas tradicionales vascas. |
| 1 | 15 | NP1-attaching | El hermano trabajaba con sus colegas. |
| 1 | 16 | NP1-attaching | El primo escribía poemas de amor. |
| 1 | 17 | NP1-attaching | El secretario patinaba con sus hijos. |
| 1 | 18 | NP1-attaching | El nieto fumaba delante del hospital. |
| 1 | 19 | NP1-attaching | El médico acosaba a su colega. |
| 1 | 20 | NP1-attaching | El cuñado nadaba en la piscina. |
| 1 | 21 | NP1-attaching | El hijo enseñaba en una escuela. |
| 2 | 22 | NP1-attaching | El cliente cantaba en el coro. |
| 2 | 23 | NP1-attaching | El abuelo silbaba como un pastor. |
| 2 | 24 | NP1-attaching | El maestro tosía por el asma. |
| 2 | 25 | NP1-attaching | El primo estudiaba en la biblioteca. |
| 2 | 26 | NP1-attaching | El cirujano salía con mi prima. |
| 2 | 27 | NP1-attaching | El tío jugaba a fútbol sala. |
| 2 | 28 | NP1-attaching | El criado corría en el parque. |
| 2 | 29 | NP1-attaching | El suegro cocinaba en la cafetería. |
| 2 | 30 | NP1-attaching | El criado comía en el comedor. |
| 2 | 31 | NP1-attaching | El abuelo conducía un viejo Seiscientos. |
| 2 | 32 | NP1-attaching | El colega caminaba con unas muletas. |
| 2 | 33 | NP1-attaching | El cocinero paseaba por el río. |
| 2 | 34 | NP1-attaching | El vecino robaba chicles del estanco. |
| 2 | 35 | NP1-attaching | El vecino bailaba danzas tradicionales vascas. |
| 2 | 36 | NP1-attaching | El nieto trabajaba con sus colegas. |
| 2 | 37 | NP1-attaching | El cuñado escribía poemas de amor. |
| 2 | 38 | NP1-attaching | El cocinero patinaba con sus hijos. |
| 2 | 39 | NP1-attaching | El sastre fumaba delante del hospital. |
| 2 | 40 | NP1-attaching | El tío acosaba a su colega. |
| 2 | 41 | NP1-attaching | El cliente nadaba en la piscina. |
| 2 | 42 | NP1-attaching | El cirujano enseñaba en una escuela. |
| 3 | 1 | NP2-attaching | El funcionario cantaba en el coro. |
| 3 | 2 | NP2-attaching | El estudiante silbaba como un pastor. |
| 3 | 3 | NP2-attaching | El bombero tosía por el asma. |
| 3 | 4 | NP2-attaching | El cantante estudiaba en la biblioteca. |
| 3 | 5 | NP2-attaching | El gobernador salía con mi prima. |
| 3 | 6 | NP2-attaching | El soldado jugaba a fútbol sala. |
| 3 | 7 | NP2-attaching | El investigador corría en el parque. |
| 3 | 8 | NP2-attaching | El joven cocinaba en la cafetería. |
| 3 | 9 | NP2-attaching | El funcionario comía en el restaurante. |
| 3 | 10 | NP2-attaching | El juez conducía un viejo Seiscientos. |
| 3 | 11 | NP2-attaching | El marqués caminaba con unas muletas. |
| 3 | 12 | NP2-attaching | El chico paseaba por el río. |
| 3 | 13 | NP2-attaching | El estudiante robaba chicles del estanco. |
| 3 | 14 | NP2-attaching | El policía bailaba danzas tradicionales vascas. |
| 3 | 15 | NP2-attaching | El bombero trabajaba con sus colegas. |
| 3 | 16 | NP2-attaching | El cazador escribía poemas de amor. |
| 3 | 17 | NP2-attaching | El director patinaba con sus hijos. |
| 3 | 18 | NP2-attaching | El comerciante fumaba delante del hospital. |
| 3 | 19 | NP2-attaching | El gobernador acosaba a su colega. |
| 3 | 20 | NP2-attaching | El cazador nadaba en la piscina. |
| 3 | 21 | NP2-attaching | El investigador enseñaba en una escuela. |
| 4 | 22 | NP2-attaching | El joven cantaba en el coro. |
| 4 | 23 | NP2-attaching | El camarero silbaba como un pastor. |
| 4 | 24 | NP2-attaching | El chico tosía por el asma. |
| 4 | 25 | NP2-attaching | El camarero estudiaba en la biblioteca. |
| 4 | 26 | NP2-attaching | El ministro salía con mi prima. |
| 4 | 27 | NP2-attaching | El músico jugaba a fútbol sala. |
| 4 | 28 | NP2-attaching | El marqués corría en el parque. |
| 4 | 29 | NP2-attaching | El especialista cocinaba en la cafetería. |
| 4 | 30 | NP2-attaching | El conde comía en el comedor. |
| 4 | 31 | NP2-attaching | El cantante conducía un viejo Seiscientos. |
| 4 | 32 | NP2-attaching | El corredor caminaba con unas muletas. |
| 4 | 33 | NP2-attaching | El conde paseaba por el río. |
| 4 | 34 | NP2-attaching | El corredor robaba chicles del estanco. |
| 4 | 35 | NP2-attaching | El soldado bailaba danzas tradicionales vascas. |
| 4 | 36 | NP2-attaching | El músico trabajaba con sus colegas. |
| 4 | 37 | NP2-attaching | El policía escribía poemas de amor. |
| 4 | 38 | NP2-attaching | El ministro patinaba con sus hijos. |
| 4 | 39 | NP2-attaching | El juez fumaba delante del hospital. |
| 4 | 40 | NP2-attaching | El especialista acosaba a su colega. |
| 4 | 41 | NP2-attaching | El comerciante nadaba en la piscina. |
| 4 | 42 | NP2-attaching | El director enseñaba en una escuela. |

**Supplementary materials for Norming Study 1: filler sentences**

| Item | Acceptability | Stimulus |
| --- | --- | --- |
| 1 | Acceptable | Ya van tres días que no puedo dormir. |
| 2 | Acceptable | Mi padre va al psicólogo cada semana. |
| 3 | Acceptable | La presentadora lleva un vestido verde. |
| 4 | Unacceptable | A mi gata las encanta dar paseos. |
| 5 | Unacceptable | El novio se maltrata por la madre. |
| 6 | Unacceptable | Las guerrilleras fermaron el fuego. |
| 1 | Acceptable | Esta es mi habitación. |
| 2 | Acceptable | Mi amigo imparte clases de costura. |
| 3 | Acceptable | Mi compañero de piso tiene la música alta. |
| 4 | Acceptable | Julio quisiera ir al cine. |
| 5 | Acceptable | El sábado votaremos para las elecciones. |
| 6 | Acceptable | Este trabajo es una muy buena oportunidad. |
| 7 | Acceptable | El dueño del piso es un buen hombre. |
| 8 | Acceptable | El peluquero hizo una estancia de trabajo en Londres. |
| 9 | Acceptable | A todos les gusta el helado. |
| 10 | Acceptable | El viudo está muy afligido. |
| 11 | Acceptable | La hija sacó el carnet de conducir. |
| 12 | Acceptable | La novia toca en un grupo de rock. |
| 13 | Acceptable | Marisa está perdidamente enamorada de Emily. |
| 14 | Acceptable | La profesora es muy asertiva. |
| 15 | Acceptable | Cambiarán el nombre de esta calle. |
| 16 | Acceptable | Estoy segura de que llegaremos a un acuerdo. |
| 17 | Acceptable | Me gusta mucho hacer papiroflexia. |
| 18 | Acceptable | En septiembre empezará la universidad. |
| 19 | Acceptable | El año que viene me mudaré a Holanda. |
| 20 | Acceptable | He tenido una discusión intensa con mi madre. |
| 21 | Acceptable | Mi novio trabaja de enfermero. |
| 1 | Unacceptable | El marqueses no amaba a los animales. |
| 2 | Unacceptable | Estimo mucho a la entrevistador de Telecinco. |
| 3 | Unacceptable | Los jefas de Alvaro eran muy estrictas. |
| 4 | Unacceptable | Mi amiga Ángeles no café bebe. |
| 5 | Unacceptable | Es muy bonito el abrigo que te has comprada. |
| 6 | Unacceptable | Josefa ha empujado que no le hacía caso. |
| 7 | Unacceptable | Esther coge una florero. |
| 8 | Unacceptable | Vimos a Gonzalo y que siempre se me olvidó. |
| 9 | Unacceptable | La fiesta se atrasará hasta el finde pasado. |
| 10 | Unacceptable | Sonia tenió un novio de Andalucía. |
| 11 | Unacceptable | Está la calle cerrado al paso. |
| 12 | Unacceptable | Isabel y yo vivo en el casco viejo. |
| 13 | Unacceptable | Dejé los cascos que me compraste en la despacho. |
| 14 | Unacceptable | Tomás celebraron su compleaños solo en casa. |
| 15 | Unacceptable | A mi gato le gustaba mirar por los ventana. |
| 16 | Unacceptable | Viviremos en Estocolmo durante muchos escritorios. |
| 17 | Unacceptable | El nene tiene mucha hambra. |
| 18 | Unacceptable | El pantalla se rompió ayer a la tarde. |
| 19 | Unacceptable | Mi jefa se mosqueó para haber llegado tarde. |
| 20 | Unacceptable | La profesoresa me hizo una pregunta. |
| 21 | Unacceptable | La baterística suena muy muy bien. |
| 22 | Unacceptable | La tennista cantaba el himno. |
| 23 | Unacceptable | El barman servía clavos por las mesas. |
| 24 | Unacceptable | El perdente se quedó triste tras la competición. |
| 25 | Unacceptable | El tendero hizo los saltos por la felicidad. |
| 26 | Unacceptable | Trabajiste como masajeador el año pasado. |
| 27 | Unacceptable | Hacer ganchillo me distresa. |
| 28 | Unacceptable | La pintura pintora. |
| 29 | Unacceptable | El luthier construye quitarras todos los días. |
| 30 | Unacceptable | La consejera ridiculiza un aire cansado. |
| 31 | Unacceptable | Las libres elecsiones son un derecho fundamental. |
| 32 | Unacceptable | Acabo de volver de la ferramentería. |
| 33 | Unacceptable | Los conciertos de ayer a la noche me despertaran muy tarde. |
| 34 | Unacceptable | Estamos viviendo un memento histórico. |
| 35 | Unacceptable | Compré dos racimos de ajo. |
| 36 | Unacceptable | La administradora está en un largo reunión. |
| 37 | Unacceptable | La bebé cayóse del carrusel. |
| 38 | Unacceptable | Tienes una manchia de tomate en el jersey. |
| 39 | Unacceptable | La homicidia de Kennedy fue un acontecimiento abrumador. |
| 40 | Unacceptable | La hija del surtidora estudia en Oxford. |
| 41 | Unacceptable | Tengo una amiga muy pardo. |
| 42 | Unacceptable | A la señores les gusta viajar por trabajo. |

**Supplementary materials for Norming Study 2: experimental sentences**

| List | Item | Stimulus |
| --- | --- | --- |
| 1 | 1 | Entreví a David que corría en el parque. |
| 1 | 2 | Cotilleé a Manuel que patinaba con sus amigas. |
| 1 | 3 | Vigilé a Marisa que jugaba a futbol. |
| 1 | 4 | Pillé a Irene que esquiaba por la pista. |
| 1 | 5 | Miré a Borja que caminaba por el río. |
| 1 | 6 | Vislumbré a Inés que lloraba por el duelo. |
| 1 | 7 | Retraté a Paco que fumaba un puro cubano. |
| 1 | 8 | Dibujé a Antonio que jugaba con el cachorro. |
| 1 | 9 | Espié a Javier que fregaba los platos. |
| 1 | 10 | Imaginé a Pablo que entrenaba para la competición. |
| 1 | 11 | Descubrí a Teresa que tocaba el saxophone. |
| 1 | 12 | Oí a Luis que tocaba la guitarra. |
| 1 | 13 | Percibí a Rocío que cantaba mi canción favorita. |
| 1 | 14 | Entreoí a Isabel que tocaba el piano. |
| 2 | 15 | Noté a Carlos que corría en el parque. |
| 2 | 16 | Reconocí a Juan que patinaba con sus amigas. |
| 2 | 17 | Vi a Marta que jugaba a futbol. |
| 2 | 18 | Estudié a Rosa que esquiaba por la pista. |
| 2 | 19 | Soñé con Paola que caminaba por el río. |
| 2 | 20 | Observé a Javier que lloraba por el duelo. |
| 2 | 21 | Delineé a Pedro que fumaba un puro cubano. |
| 2 | 22 | Atisbé a Marisol que jugaba con el cachorro. |
| 2 | 23 | Contemplé a Marisol que fregaba los platos. |
| 2 | 24 | Fotografié a Isabel que entrenaba para la competición. |
| 2 | 25 | Sorprendí a Alberto que tocaba el saxophone. |
| 2 | 26 | Sentí a María que tocaba la guitarra. |
| 2 | 27 | Escuché a Sergio que cantaba mi canción favorita. |
| 2 | 28 | Grabé a Beatriz que tocaba el piano. |

**Supplementary materials for Norming Study 2: filler sentences**

| Item | Acceptability | Stimulus |
| --- | --- | --- |
| Train | Acceptable | Tengo el ordenador roto. |
| Train | Acceptable | El chico del gorro está muy guapo. |
| Train | Acceptable | Tengo un gato muy torpe. |
| Train | Unacceptable | El pingüínos es un animal muy elegante. |
| Train | Unacceptable | El año pasado leí unas libros de Bernardo Atxaga. |
| Train | Unacceptable | Nuestra pelicula feverita es Siete Apellidos Vascos. |
| 1 | Acceptable | Nunca he estado en Alemania. |
| 2 | Acceptable | Lorena le escribió una carta al concejal. |
| 3 | Acceptable | He comido con Gregorio al mediodía. |
| 4 | Acceptable | Mi hermano trabaja de modelo. |
| 5 | Acceptable | José Luis es muy gracioso. |
| 6 | Acceptable | Conocemos a ese señor que lleva sandalias. |
| 7 | Acceptable | César se lastimó un tobillo. |
| 8 | Acceptable | No traje bombones para ti. |
| 9 | Acceptable | Felipe lloraba como un crío. |
| 10 | Acceptable | Ismael es un chico muy majo de Zaragoza. |
| 11 | Acceptable | El verano pasado estuve en Cáceres. |
| 12 | Acceptable | Dile a Ignacio que tiene los cordones sueltos. |
| 13 | Acceptable | No soporto a Hector y a su novia. |
| 14 | Acceptable | Me contaron que su abuela se había muerto. |
| 15 | Acceptable | Samuel no tiene la culpa. |
| 16 | Acceptable | Espero que te lo pases bien de vacaciones. |
| 17 | Acceptable | Ése es el profesor que viene al gimnasio conmigo. |
| 18 | Acceptable | Fidel Castro fue un presidente cubano. |
| 19 | Acceptable | Falleció ayer el niño que tenía leucemia. |
| 20 | Acceptable | Los sindicatos convocaron una huelga. |
| 21 | Acceptable | El periodista publicó un libro muy interesante. |
| 1 | Unacceptable | Picasso ha pintó este cuadro. |
| 2 | Unacceptable | He avisaba a Paco. |
| 3 | Unacceptable | Nuestro perro amabas los calcetines. |
| 4 | Unacceptable | El alcalde peatonalizó al vecino. |
| 5 | Unacceptable | La modista creí a Begoña que paseaba. |
| 6 | Unacceptable | Vivo con el chica que bostezaba. |
| 7 | Unacceptable | El jovencito amistaba a sus pantalones. |
| 8 | Unacceptable | El zapato no me cabo. |
| 9 | Unacceptable | El ordenador agobiamos al hermano. |
| 10 | Unacceptable | Cotejé a Asunción. |
| 11 | Unacceptable | El señorito jadeó al cotilleo. |
| 12 | Unacceptable | La peluquera no gustaba su perro. |
| 13 | Unacceptable | El alienigena corro y se tumbó en el suelo. |
| 14 | Unacceptable | La gata se zampé todos los bollos. |
| 15 | Unacceptable | Avisé a la vecina que no corbata gris. |
| 16 | Unacceptable | La secretaria de Rafael han muerto. |
| 17 | Unacceptable | El paleontólogo quedaba a la enfermera que saltaba. |
| 18 | Unacceptable | La chica del colegio ha estada enferma. |
| 19 | Unacceptable | Soy licenciata en derecho. |
| 20 | Unacceptable | La arquitecta pensaba a sus hijos. |
| 21 | Unacceptable | Yo ayer se fueron al gimnasio. |

**Supplementary materials for Experiments 1a and 1b: experimental sentences**

| Item | Matrix verb | Attachment | Stimulus |
| --- | --- | --- | --- |
| 1 | Perceptual | Low | María escuchó al hijo de los funcionarios que cantaban en el coro. |
| 1 | Perceptual | High | María escuchó a los hijos del funcionario que cantaban en el coro. |
| 1 | Non-perceptual | Low | María entrenó al hijo de los funcionarios que cantaban en el coro. |
| 1 | Non-perceptual | High | María entrenó a los hijos del funcionario que cantaban en el coro. |
| 2 | Perceptual | Low | Teresa miró al jefe de los bomberos que tosían por el asma. |
| 2 | Perceptual | High | Teresa miró a los jefes del bombero que tosían por el asma. |
| 2 | Non-perceptual | Low | Teresa esperó al jefe de los bomberos que tosían por el asma. |
| 2 | Non-perceptual | High | Teresa esperó a los jefes del bombero que tosían por el asma. |
| 3 | Perceptual | Low | Luis contempló al niño de los cantantes que estudiaban en la biblioteca. |
| 3 | Perceptual | High | Luis contempló a los niños del cantante que estudiaban en la biblioteca. |
| 3 | Non-perceptual | Low | Luis ayudó al niño de los cantantes que estudiaban en la biblioteca. |
| 3 | Non-perceptual | High | Luis ayudó a los niños del cantante que estudiaban en la biblioteca. |
| 4 | Perceptual | Low | Alberto atisbó al colega de los gobernadores que salían con mi prima. |
| 4 | Perceptual | High | Alberto atisbó a los colegas del gobernador que salían con mi prima. |
| 4 | Non-perceptual | Low | Alberto envidió al colega de los gobernadores que salían con mi prima. |
| 4 | Non-perceptual | High | Alberto envidió a los colegas del gobernador que salían con mi prima. |
| 5 | Perceptual | Low | Amparo fotografió al médico de los soldados que jugaban a fútbol sala. |
| 5 | Perceptual | High | Amparo fotografió a los médicos del soldado que jugaban a fútbol sala. |
| 5 | Non-perceptual | Low | Amparo señaló al médico de los soldados que jugaban a fútbol sala. |
| 5 | Non-perceptual | High | Amparo señaló a los médicos del soldado que jugaban a fútbol sala. |
| 6 | Perceptual | Low | Sergio reconoció al niño de los investigadores que corrían en el parque. |
| 6 | Perceptual | High | Sergio reconoció a los niños del investigador que corrían en el parque. |
| 6 | Non-perceptual | Low | Sergio regañó al niño de los investigadores que corrían en el parque. |
| 6 | Non-perceptual | High | Sergio regañó a los niños del investigador que corrían en el parque. |
| 7 | Perceptual | Low | Rocío vio al maestro de los jóvenes que cocinaban en la cafetería. |
| 7 | Perceptual | High | Rocío vio a los maestros del joven que cocinaban en la cafetería. |
| 7 | Non-perceptual | Low | Rocío llamó al maestro de los jóvenes que cocinaban en la cafetería. |
| 7 | Non-perceptual | High | Rocío llamó a los maestros del joven que cocinaban en la cafetería. |
| 8 | Perceptual | Low | Beatriz observó al jefe de los funcionarios que comían en el restaurante. |
| 8 | Perceptual | High | Beatriz observó a los jefes del funcionario que comían en el restaurante. |
| 8 | Non-perceptual | Low | Beatriz abrazó al jefe de los funcionarios que comían en el restaurante. |
| 8 | Non-perceptual | High | Beatriz abrazó a los jefes del funcionario que comían en el restaurante. |
| 9 | Perceptual | Low | Marta grabó al secretario de los jueces que conducían unos viejos Seiscientos. |
| 9 | Perceptual | High | Marta grabó a los secretarios del juez que conducían unos viejos Seiscientos. |
| 9 | Non-perceptual | Low | Marta dejó al secretario de los jueces que conducían unos viejos Seiscientos. |
| 9 | Non-perceptual | High | Marta dejó a los secretarios del juez que conducían unos viejos Seiscientos. |
| 10 | Perceptual | Low | Pilar vigiló al sastre de los marqueses que caminaban con unas muletas. |
| 10 | Perceptual | High | Pilar vigiló a los sastres del marqués que caminaban con unas muletas. |
| 10 | Non-perceptual | Low | Pilar visitó al sastre de los marqueses que caminaban con unas muletas. |
| 10 | Non-perceptual | High | Pilar visitó a los sastres del marqués que caminaban con unas muletas. |
| 11 | Perceptual | Low | Juan escuchó al compañero de los estudiantes que silbaban como un pastor. |
| 11 | Perceptual | High | Juan escuchó a los compañeros del estudiante que silbaban como un pastor. |
| 11 | Non-perceptual | Low | Juan entrenó al compañero de los estudiantes que silbaban como un pastor. |
| 11 | Non-perceptual | High | Juan entrenó a los compañeros del estudiante que silbaban como un pastor. |
| 12 | Perceptual | Low | José miró al suegro de los chicos que paseaban por el río. |
| 12 | Perceptual | High | José miró a los suegros del chico que paseaban por el río. |
| 12 | Non-perceptual | Low | José esperó al suegro de los chicos que paseaban por el río. |
| 12 | Non-perceptual | High | José esperó a los suegros del chico que paseaban por el río. |
| 13 | Perceptual | Low | Borja contempló al hermano de los estudiantes que robaban chicles del estanco. |
| 13 | Perceptual | High | Borja contempló a los hermanos del estudiante que robaban chicles del estanco. |
| 13 | Non-perceptual | Low | Borja ayudó al hermano de los estudiantes que robaban chicles del estanco. |
| 13 | Non-perceptual | High | Borja ayudó a los hermanos del estudiante que robaban chicles del estanco. |
| 14 | Perceptual | Low | Irene atisbó al compañero de los policías que bailaban danzas tradicionales vascas. |
| 14 | Perceptual | High | Irene atisbó a los compañeros del policía que bailaban danzas tradicionales vascas. |
| 14 | Non-perceptual | Low | Irene envidió al compañero de los policías que bailaban danzas tradicionales vascas. |
| 14 | Non-perceptual | High | Irene envidió a los compañeros del policía que bailaban danzas tradicionales vascas. |
| 15 | Perceptual | Low | Javier fotografió al primo de los cazadores que escribían poemas de amor. |
| 15 | Perceptual | High | Javier fotografió a los primos del cazador que escribían poemas de amor. |
| 15 | Non-perceptual | Low | Javier señaló al primo de los cazadores que escribían poemas de amor. |
| 15 | Non-perceptual | High | Javier señaló a los primos del cazador que escribían poemas de amor. |
| 16 | Perceptual | Low | David reconoció al secretario de los directores que patinaban con sus hijos. |
| 16 | Perceptual | High | David reconoció a los secretarios del director que patinaban con sus hijos. |
| 16 | Non-perceptual | Low | David regañó al secretario de los directores que patinaban con sus hijos. |
| 16 | Non-perceptual | High | David regañó a los secretarios del director que patinaban con sus hijos. |
| 17 | Perceptual | Low | Carmen vio al nieto de los comerciantes que fumaban delante del hospital. |
| 17 | Perceptual | High | Carmen vio a los nietos del comerciante que fumaban delante del hospital. |
| 17 | Non-perceptual | Low | Carmen llamó al nieto de los comerciantes que fumaban delante del hospital. |
| 17 | Non-perceptual | High | Carmen llamó a los nietos del comerciante que fumaban delante del hospital. |
| 18 | Perceptual | Low | Marisol observó al médico de los gobernadores que acosaban a su colega. |
| 18 | Perceptual | High | Marisol observó a los médicos del gobernador que acosaban a su colega. |
| 18 | Non-perceptual | Low | Marisol abrazó al médico de los gobernadores que acosaban a su colega. |
| 18 | Non-perceptual | High | Marisol abrazó a los médicos del gobernador que acosaban a su colega. |
| 19 | Perceptual | Low | Marijo grabó al cuñado de los cazadores que nadaban en la piscina. |
| 19 | Perceptual | High | Marijo grabó a los cuñados del cazador que nadaban en la piscina. |
| 19 | Non-perceptual | Low | Marijo dejó al cuñado de los cazadores que nadaban en la piscina. |
| 19 | Non-perceptual | High | Marijo dejó a los cuñados del cazador que nadaban en la piscina. |
| 20 | Perceptual | Low | Ana vigiló al hijo de los investigadores que enseñaban en una escuela. |
| 20 | Perceptual | High | Ana vigiló a los hijos del investigador que enseñaban en una escuela. |
| 20 | Non-perceptual | Low | Ana visitó al hijo de los investigadores que enseñaban en una escuela. |
| 20 | Non-perceptual | High | Ana visitó a los hijos del investigador que enseñaban en una escuela. |
| 21 | Perceptual | Low | Isabel escuchó al cliente de los jóvenes que cantaban en el coro. |
| 21 | Perceptual | High | Isabel escuchó a los clientes del joven que cantaban en el coro. |
| 21 | Non-perceptual | Low | Isabel entrenó al cliente de los jóvenes que cantaban en el coro. |
| 21 | Non-perceptual | High | Isabel entrenó a los clientes del joven que cantaban en el coro. |
| 22 | Perceptual | Low | Antonio miró al maestro de los chicos que tosían por el asma. |
| 22 | Perceptual | High | Antonio miró a los maestros del chico que tosían por el asma. |
| 22 | Non-perceptual | Low | Antonio esperó al maestro de los chicos que tosían por el asma. |
| 22 | Non-perceptual | High | Antonio esperó a los maestros del chico que tosían por el asma. |
| 23 | Perceptual | Low | Paco contempló al primo de los camareros que estudiaban en la biblioteca. |
| 23 | Perceptual | High | Paco contempló a los primos del camarero que estudiaban en la biblioteca. |
| 23 | Non-perceptual | Low | Paco ayudó al primo de los camareros que estudiaban en la biblioteca. |
| 23 | Non-perceptual | High | Paco ayudó a los primos del camarero que estudiaban en la biblioteca. |
| 24 | Perceptual | Low | Pablo atisbó al cirujano de los ministros que salían con mi prima. |
| 24 | Perceptual | High | Pablo atisbó a los cirujanos del ministro que salían con mi prima. |
| 24 | Non-perceptual | Low | Pablo envidió al cirujano de los ministros que salían con mi prima. |
| 24 | Non-perceptual | High | Pablo envidió a los cirujanos del ministro que salían con mi prima. |
| 25 | Perceptual | Low | Pedro fotografió al tío de los músicos que jugaban a fútbol sala. |
| 25 | Perceptual | High | Pedro fotografió a los tíos del músico que jugaban a fútbol sala. |
| 25 | Non-perceptual | Low | Pedro señaló al tío de los músicos que jugaban a fútbol sala. |
| 25 | Non-perceptual | High | Pedro señaló a los tíos del músico que jugaban a fútbol sala. |
| 26 | Perceptual | Low | Carlos reconoció al criado de los marqueses que corrían en el parque. |
| 26 | Perceptual | High | Carlos reconoció a los criados del marqués que corrían en el parque. |
| 26 | Non-perceptual | Low | Carlos regañó al criado de los marqueses que corrían en el parque. |
| 26 | Non-perceptual | High | Carlos regañó a los criados del marqués que corrían en el parque. |
| 27 | Perceptual | Low | Jesús vio al suegro de los especialistas que cocinaban en la cafetería. |
| 27 | Perceptual | High | Jesús vio a los suegros del especialista que cocinaban en la cafetería. |
| 27 | Non-perceptual | Low | Jesús llamó al suegro de los especialistas que cocinaban en la cafetería. |
| 27 | Non-perceptual | High | Jesús llamó a los suegros del especialista que cocinaban en la cafetería. |
| 28 | Perceptual | Low | Rafael observó al criado de los condes que comían en el comedor. |
| 28 | Perceptual | High | Rafael observó a los criados del conde que comían en el comedor. |
| 28 | Non-perceptual | Low | Rafael abrazó al criado de los condes que comían en el comedor. |
| 28 | Non-perceptual | High | Rafael abrazó a los criados del conde que comían en el comedor. |
| 29 | Perceptual | Low | Miguel grabó al abuelo de los cantantes que conducían unos viejos Seiscientos. |
| 29 | Perceptual | High | Miguel grabó a los abuelos del cantante que conducían unos viejos Seiscientos. |
| 29 | Non-perceptual | Low | Miguel dejó al abuelo de los cantantes que conducían unos viejos Seiscientos. |
| 29 | Non-perceptual | High | Miguel dejó a los abuelos del cantante que conducían unos viejos Seiscientos. |
| 30 | Perceptual | Low | Elena vigiló al colega de los corredores que caminaban con unas muletas. |
| 30 | Perceptual | High | Elena vigiló a los colegas del corredor que caminaban con unas muletas. |
| 30 | Non-perceptual | Low | Elena visitó al colega de los corredores que caminaban con unas muletas. |
| 30 | Non-perceptual | High | Elena visitó a los colegas del corredor que caminaban con unas muletas. |
| 31 | Perceptual | Low | Manuel escuchó al abuelo de los camareros que silbaban como un pastor. |
| 31 | Perceptual | High | Manuel escuchó a los abuelos del camarero que silbaban como un pastor. |
| 31 | Non-perceptual | Low | Manuel entrenó al abuelo de los camareros que silbaban como un pastor. |
| 31 | Non-perceptual | High | Manuel entrenó a los abuelos del camarero que silbaban como un pastor. |
| 32 | Perceptual | Low | Nuria miró al cocinero de los condes que paseaban por el río. |
| 32 | Perceptual | High | Nuria miró a los cocineros del conde que paseaban por el río. |
| 32 | Non-perceptual | Low | Nuria esperó al cocinero de los condes que paseaban por el río. |
| 32 | Non-perceptual | High | Nuria esperó a los cocineros del conde que paseaban por el río. |
| 33 | Perceptual | Low | Josefa contempló al vecino de los corredores que robaban chicles del estanco. |
| 33 | Perceptual | High | Josefa contempló a los vecinos del corredor que robaban chicles del estanco. |
| 33 | Non-perceptual | Low | Josefa ayudó al vecino de los corredores que robaban chicles del estanco. |
| 33 | Non-perceptual | High | Josefa ayudó a los vecinos del corredor que robaban chicles del estanco. |
| 34 | Perceptual | Low | Cristina atisbó al vecino de los soldados que bailaban danzas tradicionales vascas. |
| 34 | Perceptual | High | Cristina atisbó a los vecinos del soldado que bailaban danzas tradicionales vascas. |
| 34 | Non-perceptual | Low | Cristina envidió al vecino de los soldados que bailaban danzas tradicionales vascas. |
| 34 | Non-perceptual | High | Cristina envidió a los vecinos del soldado que bailaban danzas tradicionales vascas. |
| 35 | Perceptual | Low | Ángeles fotografió al nieto de los músicos que trabajaban con sus colegas. |
| 35 | Perceptual | High | Ángeles fotografió a los primos del músico que trabajaban con sus colegas. |
| 35 | Non-perceptual | Low | Ángeles señaló al nieto de los músicos que trabajaban con sus colegas. |
| 35 | Non-perceptual | High | Ángeles señaló a los primos del músico que trabajaban con sus colegas. |
| 36 | Perceptual | Low | Laura reconoció al cuñado de los policías que escribían poemas de amor. |
| 36 | Perceptual | High | Laura reconoció a los cuñados del policía que escribían poemas de amor. |
| 36 | Non-perceptual | Low | Laura regañó al cuñado de los policías que escribían poemas de amor. |
| 36 | Non-perceptual | High | Laura regañó a los cuñados del policía que escribían poemas de amor. |
| 37 | Perceptual | Low | Sofía vio al cocinero de los ministros que patinaban con sus hijos. |
| 37 | Perceptual | High | Sofía vio a los cocineros del ministro que patinaban con sus hijos. |
| 37 | Non-perceptual | Low | Sofía llamó al cocinero de los ministros que patinaban con sus hijos. |
| 37 | Non-perceptual | High | Sofía llamó a los cocineros del ministro que patinaban con sus hijos. |
| 38 | Perceptual | Low | Julia observó al sastre de los jueces que fumaban delante del hospital. |
| 38 | Perceptual | High | Julia observó a los sastres del juez que fumaban delante del hospital. |
| 38 | Non-perceptual | Low | Julia abrazó al sastre de los jueces que fumaban delante del hospital. |
| 38 | Non-perceptual | High | Julia abrazó a los sastres del juez que fumaban delante del hospital. |
| 39 | Perceptual | Low | Lara grabó al cliente de los comerciantes que nadaban en la piscina. |
| 39 | Perceptual | High | Lara grabó a los clientes del comerciante que nadaban en la piscina. |
| 39 | Non-perceptual | Low | Lara dejó al cliente de los comerciantes que nadaban en la piscina. |
| 39 | Non-perceptual | High | Lara dejó a los clientes del comerciante que nadaban en la piscina. |
| 40 | Perceptual | Low | Francisco vigiló al cirujano de los directores que enseñaban en una escuela. |
| 40 | Perceptual | High | Francisco vigiló a los cirujanos del director que enseñaban en una escuela. |
| 40 | Non-perceptual | Low | Francisco visitó al cirujano de los directores que enseñaban en una escuela. |
| 40 | Non-perceptual | High | Francisco visitó a los cirujanos del director que enseñaban en una escuela. |

**Supplementary materials for Experiments 2a and 2b: experimental sentences**

| Item | Matrix verb | Stimulus |
| --- | --- | --- |
| 1 | Perceptual | María escuchó al hijo del funcionario que cantaba en el coro. |
| 1 | Non-perceptual | María entrenó al hijo del funcionario que cantaba en el coro. |
| 2 | Perceptual | Teresa miró al jefe del bombero que tosía por el asma. |
| 2 | Non-perceptual | Teresa esperó al jefe del bombero que tosía por el asma. |
| 3 | Perceptual | Luis contempló al niño del cantante que estudiaba en la biblioteca. |
| 3 | Non-perceptual | Luis ayudó al niño del cantante que estudiaba en la biblioteca. |
| 4 | Perceptual | Alberto atisbó al colega del gobernador que salía con mi prima. |
| 4 | Non-perceptual | Alberto envidió al colega del gobernador que salía con mi prima. |
| 5 | Perceptual | Amparo fotografió al médico del soldado que jugaba a fútbol sala. |
| 5 | Non-perceptual | Amparo señaló al médico del soldado que jugaba a fútbol sala. |
| 6 | Perceptual | Sergio reconoció al niño del investigador que corría en el parque. |
| 6 | Non-perceptual | Sergio regañó al niño del investigador que corría en el parque. |
| 7 | Perceptual | Rocío vio al maestro del joven que cocinaba en la cafetería. |
| 7 | Non-perceptual | Rocío llamó al maestro del joven que cocinaba en la cafetería. |
| 8 | Perceptual | Beatriz observó al jefe del funcionario que comía en el restaurante. |
| 8 | Non-perceptual | Beatriz abrazó al jefe del funcionario que comía en el restaurante. |
| 9 | Perceptual | Marta grabó al secretario del juez que conducía un viejo Seiscientos. |
| 9 | Non-perceptual | Marta dejó al secretario del juez que conducía un viejo Seiscientos. |
| 10 | Perceptual | Pilar vigiló al sastre del marqués que caminaba con unas muletas. |
| 10 | Non-perceptual | Pilar visitó al sastre del marqués que caminaba con unas muletas. |
| 11 | Perceptual | Juan escuchó al compañero del estudiante que silbaba como un pastor. |
| 11 | Non-perceptual | Juan entrenó al compañero del estudiante que silbaba como un pastor. |
| 12 | Perceptual | José miró al suegro del chico que paseaba por el río. |
| 12 | Non-perceptual | José esperó al suegro del chico que paseaba por el río. |
| 13 | Perceptual | Borja contempló al hermano del estudiante que robaba chicles del estanco. |
| 13 | Non-perceptual | Borja ayudó al hermano del estudiante que robaba chicles del estanco. |
| 14 | Perceptual | Irene atisbó al compañero del policía que bailaba danzas tradicionales vascas. |
| 14 | Non-perceptual | Irene envidió al compañero del policía que bailaba danzas tradicionales vascas. |
| 15 | Perceptual | Javier fotografió al primo del cazador que escribía poemas de amor. |
| 15 | Non-perceptual | Javier señaló al primo del cazador que escribía poemas de amor. |
| 16 | Perceptual | David reconoció al secretario del director que patinaba con sus hijos. |
| 16 | Non-perceptual | David regañó al secretario del director que patinaba con sus hijos. |
| 17 | Perceptual | Carmen vio al nieto del comerciante que fumaba delante del hospital. |
| 17 | Non-perceptual | Carmen llamó al nieto del comerciante que fumaba delante del hospital. |
| 18 | Perceptual | Marisol observó al médico del gobernador que acosaba a su colega. |
| 18 | Non-perceptual | Marisol abrazó al médico del gobernador que acosaba a su colega. |
| 19 | Perceptual | Marijo grabó al cuñado del cazador que nadaba en la piscina. |
| 19 | Non-perceptual | Marijo dejó al cuñado del cazador que nadaba en la piscina. |
| 20 | Perceptual | Ana vigiló al hijo del investigador que enseñaba en una escuela. |
| 20 | Non-perceptual | Ana visitó al hijo del investigador que enseñaba en una escuela. |
| 21 | Perceptual | Isabel escuchó al cliente del joven que cantaba en el coro. |
| 21 | Non-perceptual | Isabel entrenó al cliente del joven que cantaba en el coro. |
| 22 | Perceptual | Antonio miró al maestro del chico que tosía por el asma. |
| 22 | Non-perceptual | Antonio esperó al maestro del chico que tosía por el asma. |
| 23 | Perceptual | Paco contempló al primo del camarero que estudiaba en la biblioteca. |
| 23 | Non-perceptual | Paco ayudó al primo del camarero que estudiaba en la biblioteca. |
| 24 | Perceptual | Pablo atisbó al cirujano del ministro que salía con mi prima. |
| 24 | Non-perceptual | Pablo envidió al cirujano del ministro que salía con mi prima. |
| 25 | Perceptual | Pedro fotografió al tío del músico que jugaba a fútbol sala. |
| 25 | Non-perceptual | Pedro señaló al tío del músico que jugaba a fútbol sala. |
| 26 | Perceptual | Carlos reconoció al criado del marqués que corría en el parque. |
| 26 | Non-perceptual | Carlos regañó al criado del marqués que corría en el parque. |
| 27 | Perceptual | Jesús vio al suegro del especialista que cocinaba en la cafetería. |
| 27 | Non-perceptual | Jesús llamó al suegro del especialista que cocinaba en la cafetería. |
| 28 | Perceptual | Rafael observó al criado del conde que comía en el comedor. |
| 28 | Non-perceptual | Rafael abrazó al criado del conde que comía en el comedor. |
| 29 | Perceptual | Miguel grabó al abuelo del cantante que conducía un viejo Seiscientos. |
| 29 | Non-perceptual | Miguel dejó al abuelo del cantante que conducía un viejo Seiscientos. |
| 30 | Perceptual | Elena vigiló al colega del corredor que caminaba con unas muletas. |
| 30 | Non-perceptual | Elena visitó al colega del corredor que caminaba con unas muletas. |
| 31 | Perceptual | Manuel escuchó al abuelo del camarero que silbaba como un pastor. |
| 31 | Non-perceptual | Manuel entrenó al abuelo del camarero que silbaba como un pastor. |
| 32 | Perceptual | Nuria miró al cocinero del conde que paseaba por el río. |
| 32 | Non-perceptual | Nuria esperó al cocinero del conde que paseaba por el río. |
| 33 | Perceptual | Josefa contempló al vecino del corredor que robaba chicles del estanco. |
| 33 | Non-perceptual | Josefa ayudó al vecino del corredor que robaba chicles del estanco. |
| 34 | Perceptual | Cristina atisbó al vecino del soldado que bailaba danzas tradicionales vascas. |
| 34 | Non-perceptual | Cristina envidió al vecino del soldado que bailaba danzas tradicionales vascas. |
| 35 | Perceptual | Ángeles fotografió al nieto del músico que trabajaba con sus colegas. |
| 35 | Non-perceptual | Ángeles señaló al nieto del músico que trabajaba con sus colegas. |
| 36 | Perceptual | Laura reconoció al cuñado del policía que escribía poemas de amor. |
| 36 | Non-perceptual | Laura regañó al cuñado del policía que escribía poemas de amor. |
| 37 | Perceptual | Sofía vio al cocinero del ministro que patinaba con sus hijos. |
| 37 | Non-perceptual | Sofía llamó al cocinero del ministro que patinaba con sus hijos. |
| 38 | Perceptual | Julia observó al sastre del juez que fumaba delante del hospital. |
| 38 | Non-perceptual | Julia abrazó al sastre del juez que fumaba delante del hospital. |
| 39 | Perceptual | Lara grabó al cliente del comerciante que nadaba en la piscina. |
| 39 | Non-perceptual | Lara dejó al cliente del comerciante que nadaba en la piscina. |
| 40 | Perceptual | Francisco vigiló al cirujano del director que enseñaba en una escuela. |
| 40 | Non-perceptual | Francisco visitó al cirujano del director que enseñaba en una escuela. |

**Supplementary materials for Experiments 1 and 2: filler sentences**

| Item | Stimulus |
| --- | --- |
| Train | Su sobrina estudia en la Universidad del País Vasco para ser ingeniera mecánica. |
| Train | Victoria siempre exige mucho a sus empleados porque está al mando de una empresa importante. |
| Train | Al grupo de jóvenes escritores estadounidenses de los años sesenta se les conoce como Beat Generation. |
| Train | La música jazz parece caótica pero en realidad tiene un complejo sistema de reglas armónicas. |
| Train | Sherlock Holmes es el renombradísimo investigador en los libros de Arthur Conan Doyle. |
| Train | No hay gatos a los que no les guste el contacto humano de vez en cuando. |
| 1 | Mi amiga Minori se llama así porque su familia es de Japón. |
| 2 | La música que más me gusta es el rock pero también me gusta Joaquín Sabina. |
| 3 | Francisco conoció a su novia actual gracias a Tinder. |
| 4 | Parece que han encontrado el cadáver de un hombre en los lagos de Covadonga. |
| 5 | Al periodista le faltaba el aliento porque acababa de llegar. |
| 6 | Los padres de Fátima se separaron cuando era niña. |
| 7 | El servicio de atención al cliente me dejó en espera durante un montón de tiempo. |
| 8 | El asesor de ventas de la sucursal de Toledo fue extremadamente grosero. |
| 9 | Llamo al número de apoyo técnico porque creo que la impresora se ha atascado. |
| 10 | El primer novio de mi amiga Nuria era muy irritante y soberbio. |
| 11 | Tiago es nuestro compañero portugués que se ocupa de investigación genética. |
| 12 | A día de hoy muchos chicos deciden depilarse las piernas por razones estéticas. |
| 13 | El caricaturista de ese periódico decidió dejar el puesto para dedicarse a su familia. |
| 14 | El lutier de Laura Pausini le construyó una guitarra preciosa. |
| 15 | Pedro Almodóvar fue compañero de colegio de mi padre. |
| 16 | Desde hace algunos años estudio japonés porque me gustaría ir a vivir a Kyoto. |
| 17 | Gemma tiene una relación a distancia con una universitaria de Estocolmo. |
| 18 | Pepe soñó que el año que viene Donald Trump ganará un premio Nobel. |
| 19 | Admiro muchísimo la belleza delicada de Agustín. |
| 20 | El accidente tuvo lugar en un estrecho camino del Teide. |
| 21 | Me he enterado de que la madre del restaurador se mudó a Huesca. |
| 22 | Damián persiguió al detective privado porque sospechaba de él. |
| 23 | Mi mejor amiga se ha enamorado de un becario mucho más jóven que ella. |
| 24 | Marcos nunca se pone la mascarilla por encima de la nariz. |
| 25 | Yolanda trabaja en nuestra oficina y no podríamos prescindir de ella. |
| 26 | La boda de Adrián fue realmente hermosa y conmovedora. |
| 27 | A nuestros hijos les gusta muchísimo el dibujo animado de Pixar Monstruos S.A. |
| 28 | La noticia de la hospitalización de Javier Ortega Smith se divulgó inmediatamente. |
| 29 | El barco pirata abordó el galeón español sin ninguna dificultad. |
| 30 | Muchas personas creen que la tierra es plana en vez de esférica. |
| 31 | Los huevos que Rubén compró en el mercado estaban medio podridos. |
| 32 | Después de la cena brindamos alegremente con un vaso de pacharán cada uno. |
| 33 | Es incuestionable que el hermano del bombero se haya ofendido por tus palabras. |
| 34 | El tío de Aida me contó que sufres pesadillas recurrentes. |
| 35 | Espero que hayas acudido a un especialista para que investigue este gran dolor de estómago. |
| 36 | La chica a la que molestaron el mes pasado denunció a su agresor. |
| 37 | Julián estudió inglés durante muchos años en una academia de idiomas y ahora ha obtenido una certificación. |
| 38 | Mi padre se ha cambiado de compañía telefónica hace poco y ahora está muy satisfecho con el servicio. |
| 39 | A mi gata le gusta muchísimo esconderse debajo de las mantas y fingir que está durmiendo. |
| 40 | La ponente de la conferencia de ayer también es mi profesora de física aplicada. |
| 41 | Viví durante muchos años en La Rioja porque quería trabajar de catador. |
| 42 | Dile a Juanjo que llene la botella de agua que está vacía. |
| 43 | El microondas que nos regaló mi suegra es un modelo nuevo. |
| 44 | Fuimos en coche a la boda de mis amigos y llovió durante todo el camino. |
| 45 | Este verano el aspersor automático se ha roto y se me han muerto todas las plantas. |
| 46 | Todos deberíamos llevar siempre desinfectante para cualquier eventualidad. |
| 47 | Justo cuando empezaba a llover se me ha roto el paraguas rojo. |
| 48 | Los compañeros de clase de Pelayo le han gastado una broma muy cruel. |
| 49 | La caza de brujas duró mucho más de un siglo en los Estados Unidos. |
| 50 | El viaje a Cuba fue muy estresante porque el avión era muy pequeño. |
| 51 | El presidente del consejo tropezó y se cayó durante la visita oficial en Francia. |
| 52 | Bebo mucho café porque por la mañana siempre estoy demasiado somnolienta para trabajar. |
| 53 | El nutricionista de Dolores le ha prohibido comer pasta a la hora de cenar. |
| 54 | Sufrimos muchísimo los cambios de temperatura en esta ciudad. |
| 55 | Sería genial vivir en un mundo sin religiones y sin odio. |
| 56 | Los Beatles cantaban canciones muy juveniles que hablaban sobre todo de amor. |
| 57 | El diccionario de la Real Academia Española está muy bien planteado. |
| 58 | Mi pareja es una brillante escritora de novelas policíacas. |
| 59 | Los trenes a Vitoria arrancan desde la estación de San Sebastián con una frecuencia de dos trenes por hora. |
| 60 | No envidia para nada la mala suerte de quien es alérgico al chocolate. |
| 61 | El auge del fascismo en los países europeos fue precedido por el descontento público. |
| 62 | Por fin me han llamado para esa entrevista de trabajo en el ayuntamiento. |
| 63 | Leo muchos libros pero últimamente prefiero escuchar audiolibros. |
| 64 | Nunca he visto la serie de televisión de la que me estás hablando. |
| 65 | El año pasado Tomás estuvo en una preciosa exposición de Hiroshige en Cuenca. |
| 66 | Noelia va hasta su oficina andando porque le gusta mucho pasear por el centro de Barcelona. |
| 67 | El acceso a internet que facilita la universidad siempre da algún problema de navegación. |
| 68 | Detrás de ese edificio grande y marrón está el quiosco del barrio. |
| 69 | Arturo Pérez-Reverte presentó ayer su libro en la biblioteca de Cartagena. |
| 70 | La discusión con tu novio se escuchaba incluso desde la cocina. |
| 71 | Escribí mi trabajo de fin de grado con muchísima antelación porque en julio quería irme de vacaciones. |
| 72 | La máquina que tenemos en la oficina es vieja y hace un café pésimo. |
| 73 | Los pandas están en peligro de extinción porque no pueden reproducirse en los zoos. |
| 74 | Montse quiere adoptar un pato porque su sueño es ser agricultora y granjera. |
| 75 | El traductor de Haruki Murakami ha publicado un estupendo ensayo sobre cultura japonesa. |
| 76 | El frutero de la calle Mayor estará cerrado todo el mes de Julio por vacaciones. |
| 77 | La directora preparó una buena intervención para la inauguración del año escolar. |
| 78 | Los premios Nobel de este año se los dieron a varias científicas renombradas. |
| 79 | El chico peruano que contrataron para la fiesta tiene una voz realmente bonita. |
| 80 | No conocía las novelas de la escritora galardonada el mes pasado. |
| 81 | Raúl cenó con la mujer del obrero que habían arrestado por fraude. |
| 82 | Ignacio está muy contento con la au pair porque cuida muy bien de sus hijos. |
| 83 | En las semanas de la cosecha muchos granjeros y pocos ganaderos encuentran empleo estacional. |
| 84 | Iker es animador turístico y trabaja junto a su novia que es socorrista. |
| 85 | Las amantes de esos gerentes muy distinguidos compraron el regalo de despedida. |
| 86 | Trabajé de profesor de inglés para una clase de vendedores de coches de lujo. |
| 87 | Jaime trabajó en el festival de música y conoció a muchos promotores pero sólo a un manager. |
| 88 | El patrocinador del premio literario dio un discurso conmovedor. |
| 89 | Fue el benefactor de los vagabundos el que se presentó a esa cena. |
| 90 | Manolo bebió un cóctel delicioso preparado por el asistente de sus anfitriones en Florencia. |
| 91 | La canguro de la sobrina del mecenas tiene unos ojos azules que son preciosos. |
| 92 | El monitor que había sonreído a la modista competía con su grupo de atletas. |
| 93 | Los arquitectos junto al cartero que vive al lado del colegio se quejaron de la administración municipal. |
| 94 | El escultor al que los pintores habían halagado sufrió un accidente de coche. |
| 95 | Esos autónomos antes trabajaban de artesanos del rey. |
| 96 | El albañil les reprochó a sus mecánicos de confianza un error en las facturas. |
| 97 | Me gusta mucho el logo del diseñador del bufete de abogados de mi pareja. |
| 98 | El doctorando les pidió consejo a esos científicos renombrados y ellos le ayudaron. |
| 99 | Los psicólogos no suelen tener chófer porque no tienen un sueldo estable. |
| 100 | En aquella tienda trabajan varios masajistas y un peluquero muy famoso. |
| 101 | Un señor extranjero le pidió dinero mientras los limpiaparabrisas del semáforo descansaban. |
| 102 | Los socorristas de la playa de Marbella suelen salir de fiesta con el barman de ese hotel. |
| 103 | Los granjeros se aliaron con el agricultor porque era muy amigo del alcalde. |
| 104 | El entrenador del equipo de atletas federados les mandó comprar las camisetas diseñadas por él. |
| 105 | Conozco al cartero de mi pueblo pero no conozco a su mujer. |
| 106 | Los parientes de la doctoranda le siguen preguntando qué hará de mayor y ella no sabe qué responder. |
| 107 | El dueño del estanco maltrató a sus dependientes que habían dejado escapar al ladrón. |
| 108 | Por fin el becario aprendió a ponerse la mascarilla adecuadamente gracias a la ayuda de su enamorada. |
| 109 | Óscar se percató de que los familiares del biólogo le estaban tomando el pelo. |
| 110 | Los esgrimistas se enfadaron mucho con los técnicos del ayuntamiento por su incompetencia. |
| 111 | Andrés se percató de que el esgrimista les hacía muecas a las mujeres que pasaban por la calle. |
| 112 | Josema ama a la madre de los dos actores mellizos que fueron galardonados el año pasado. |
| 113 | El astrólogo estima al astrónomo pero el astrónomo odia a todos los astrólogos. |
| 114 | El fiscal de Almería tiene una relación clandestina con una abogada casada con tres hijos. |
| 115 | Los dentistas le alquilan un despacho al fisioterapeuta porque andan justos de dinero. |
| 116 | El ahijado del fotógrafo que trabajó en mi boda es un chaval muy tímido y correcto. |
| 117 | Los padrinos de la boda le pidieron al pescadero que no viniera. |
| 118 | El torero presumido les escupió a los repartidores de pizzas mientras le pasaban al lado. |
| 119 | El mago de Disneylandia obtuvo el apoyo de los payasos y montó un sindicato. |
| 120 | La madre de Alejandro se casó con un cirujano aun teniendo a muchos otros pretendientes. |
